# Global trade drives transboundary transfer of the health impacts of polycyclic aromatic hydrocarbon emissions

**DOI:** 10.1038/s43247-022-00500-y

**Published:** 2022-08-01

**Authors:** Ruifei Li, Jin Zhang, Peter Krebs

**Affiliations:** 1 Institute of Urban and Industrial Water Management, Technische Universität Dresden, 01069 Dresden, Germany; 2 State Key Laboratory of Hydrology-Water Resources and Hydraulic Engineering, Hohai University, 210098 Nanjing, China; 3 Yangtze Institute for Conservation and Development, Hohai University, 210098 Nanjing, China

**Keywords:** Environmental impact, Environmental impact, Environmental economics, Socioeconomic scenarios

## Abstract

International trade leads to a redistribution of pollutant emissions related to the production of goods and services and subsequently affects their severe health impacts. Here, we present a framework of emissions inventories, input-output model, numerical atmospheric chemistry model, and estimates of the global burden of disease. Specifically, we assess emissions and health impacts of polycyclic aromatic hydrocarbons (PAH), a carcinogenic byproduct of production activities, and consider income, production, final sale, and consumption stages of the global supply chain between 2012 and 2015. We find that in 2015, global anthropogenic PAH emissions were 304 Gg (95% CI: 213~421 Gg) and estimated related lifetime lung cancer deaths were 6.9 × 10^4^ (95% CI: 1.8 × 10^4^~1.5 × 10^5^ deaths). The role of trade in driving the PAH-related health risks was greater than that in driving the emissions. Our findings indicate that international cooperation is needed to optimise the global supply chains and mitigate PAH emissions and health impacts.

## Abstract (web summary)

International trade affects polycyclic aromatic hydrocarbon emissions and their health impacts attributed to different stages of global supply chains, according to a framework of emission inventories and numerical models.

## Introduction

Outdoor air pollution and its associated health impacts have been regarded as important global concerns^1,2^. It has been reported that more than 8.42 million premature deaths occurred due to human exposure to ambient particulate matter in 2016 worldwide^3,4^. As one of the most toxic persistent organic compound groups, polycyclic aromatic hydrocarbons (PAHs) have been proved to be mutagenic, carcinogenic, and teratogenic, and have negative effects on the immune system development, host resistance, and humoral immunity, even at low exposure levels^5–7^. Many previous studies have reported the increased lung cancer risks from both occupational and environmental exposure to PAHs^8–10^. Due to their widespread occurrence and toxic effects on ecological safety and human health, PAHs are involved in the Convention on Long-Range Transboundary Air Pollution Protocol^11,12^. Therefore, there is an urgent need to prevent the health burden of PAH exposure through the development of global mitigation strategies.

The majority of atmospheric PAH emissions originate as a byproduct of combustions in various economic activities^13^. Over the last few decades, worldwide economic sectors have been connected by globalization and international trade, boosting global economic growth. However, international trade redistributes emissions related to the production of goods and services and subsequently affects global air pollution and associated health impacts. Since PAH emissions are released during production activities in most economic sectors^13^, PAH-related health impacts are strongly associated with globalization and international trade. Therefore, the PAH outdoor exposure is no longer a regional problem, but a global concern due to the long-distance atmospheric pollutant transport and redistributing emissions derived from international trade^3,14^. Although the international trade-derived emissions of CO_2_ and PM_2.5_ and associated adverse health effects have been reported^3,15^, the global PAH emissions and transboundary health impacts attributed to the international trade are still largely unknown. The current separation between PAH pollution and the supply chain can greatly undermine local efforts on mitigating emissions and related health impacts in the world.

From the perspective of international trade, most previous studies analyzed consumption-based emissions and health impacts to reveal the consumers’ environmental responsibility at global and national levels^16,17^. Generally, the production process requires financial support for primary inputs and sales of final products. Primary suppliers could receive a payment from the inputs, and final sellers’ profit from the sales of final products so that they should also take the environmental duty of emissions and health impacts during the production. Therefore, the primary suppliers and final sellers play crucial roles in the global supply chain, as well as transferring the pollution^18,19^. However, less attention has been paid to quantifying the income-based and final sale-based emissions, which are important to mitigate pollution from a supply chain perspective^20,21^.

Temporal changes in PAH emissions and health impacts can be greatly influenced by many socioeconomic determinants and environmental factors. PAH emissions were estimated to be decreasing^13^, but the trends and drivers of PAH-related health impacts are largely unknown. Socioeconomic drivers mainly affect the amount of emissions through international trade, such as production structure and final demand level. Previous studies have investigated the influences of socioeconomic drivers on various emissions. For instance, it is reported that energy consumption structure was identified as the main factor impacting PM_2.5_ pollution in developing countries^22^. Changes in production structure and efficiency gains caused the decline in CO_2_ emissions embodied in exports^23^. Primary input and final demand structures had strong impacts on PAH emissions^24^. In addition, environmental factors mainly influence the atmospheric transportation of pollution, especially for PAHs, which tend to be particle-band. It has been found that meteorological changes can drive the increase in global air pollution and their related deaths in the recent future^25^, and the warming in the Arctic enabled PAH concentration to increase in the Arctic atmosphere^26^. However, optimizing the global mitigation strategies urgently required the quantification of the contributions of both socioeconomic and environmental drivers to the changes in global health impacts attributed to PAH pollution. Thus, it is vital to gain a deep understanding of the drivers of the changes in PAH emissions and health impacts for policy decision-makers to optimize mitigation strategies from the global supply chain perspective.

Consequently, this study applied an integrated framework, combining global PAH emission inventory, environmentally extended multi-regional input-output (EE-MRIO) model, GEOS-Chem chemical transport model, lifetime lung cancer risk assessment, and structural decomposition analysis (SDA), to investigate the contributions of driving forces to the PAH emissions and health impacts from whole stages of the economic supply chain (Supplementary Figs. 1, 2). The global PAH emission inventories were generated in this study based on the data of emission factors, fuel consumption, and industrial processes (Supplementary Notes 1, 2, Supplementary Data 1, 2, Supplementary Tables 1, 2). The EE-MRIO model allows for emitted emissions to be tied to each region’s investment, final sales, and consumer purchasing behaviors (Supplementary Note 3, Supplementary Table 3). Then, the income-, production-, final sale-, and consumption-based PAH emissions of 13 worldwide regions from 2012 to 2015 were determined through PAH emission inventories and the EE-MRIO model. GEOS-Chem chemical transport model was applied to simulate the concentrations in different scenarios (emission maps of different regions from a multi-perspective). The lifetime lung cancer risk was estimated according to the simulated scenarios-specific concentrations and the Global Burden of Disease (GBD) database. Finally, the contributions of different drivers to the temporal changes in the emissions and health impacts were quantified by combining structural decomposition analysis with the above models. The results of this study can assist policy decisions to optimize mitigation strategies from different perspectives of economic supply chains for effectively reducing PAH emissions and health impacts in the world.

## Results

### PAH emissions and health impacts based on global supply chain

The global PAH emissions in 2015 were 357 Gg (anthropogenic sources: 304 Gg, 95% CI: 213~421 Gg). The lifetime lung cancer deaths caused by exposure to PAH pollution were estimated as 6.9 × 10^4^ (95% CI: 1.8 × 10^4^~1.5 × 10^5^ deaths). Approximately 85% of emissions and 99% of deaths were attributed to anthropogenic activities.

**Fig. 1:**
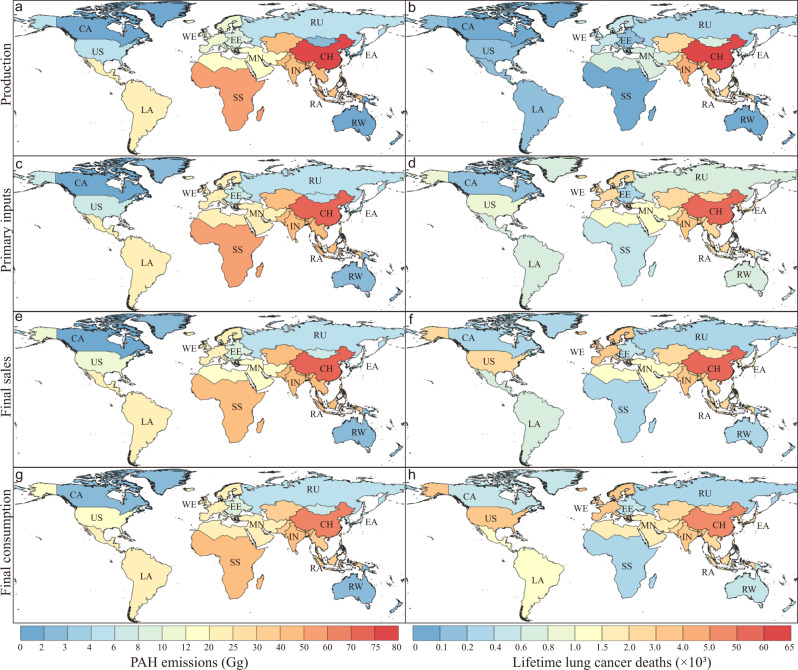
The global PAH emissions and associated health impacts in 2015 from multi-perspectives. The PAH emissions and PAH-related lifetime lung cancer deaths in 13 worldwide regions in 2015 attributed to each region’s (**a**, **b**) production, **c**, **d** primary inputs, **e**, **f** final sales, and **g**, **h** final consumption. CA Canada, CN China, EE Eastern Europe, IN India, LA Latin America, MN Middle East and north Africa, RA Rest of Asia, RE Rest of east Asia, RW Rest of the world, RU Russia, SS Sub-Saharan Africa, US the USA, WE Western Europe.

The huge differences in PAH emissions and health impacts between regions can be observed. Figure 1 shows the PAH emissions and lifetime lung cancer deaths caused by each region’s production, primary inputs, final sales, and final consumption in 2015. From the perspective of production, the emissions and deaths of developing regions, including China, India, and the rest of Asia were larger than the developed regions, such as the USA, western Europe, and East Asia. For instance, China had the largest values of emissions (78 Gg) and deaths (6.0 × 10^4^ deaths) caused by its production (Fig. 1a, b), which was attributed to its role in the global supply chain and dense population. From the perspective of the supply chain, the emissions and deaths caused by the primary inputs, final sales, and final consumption of developed regions were larger than those due to their production. For example, the production-based emissions of western Europe were 11.5 Gg, resulting in 5.0 × 10^2^ lifetime lung cancer deaths (Fig. 1a, b), while 3.8 × 10^3^ deaths were related to its consumption-based emissions (22.8 Gg) (Fig. 1g, h). The results reveal that developed regions transferred PAH emissions and related health risks to other regions through international trade as the roles of primary suppliers, final sellers, and final consumers.

**Fig. 2:**
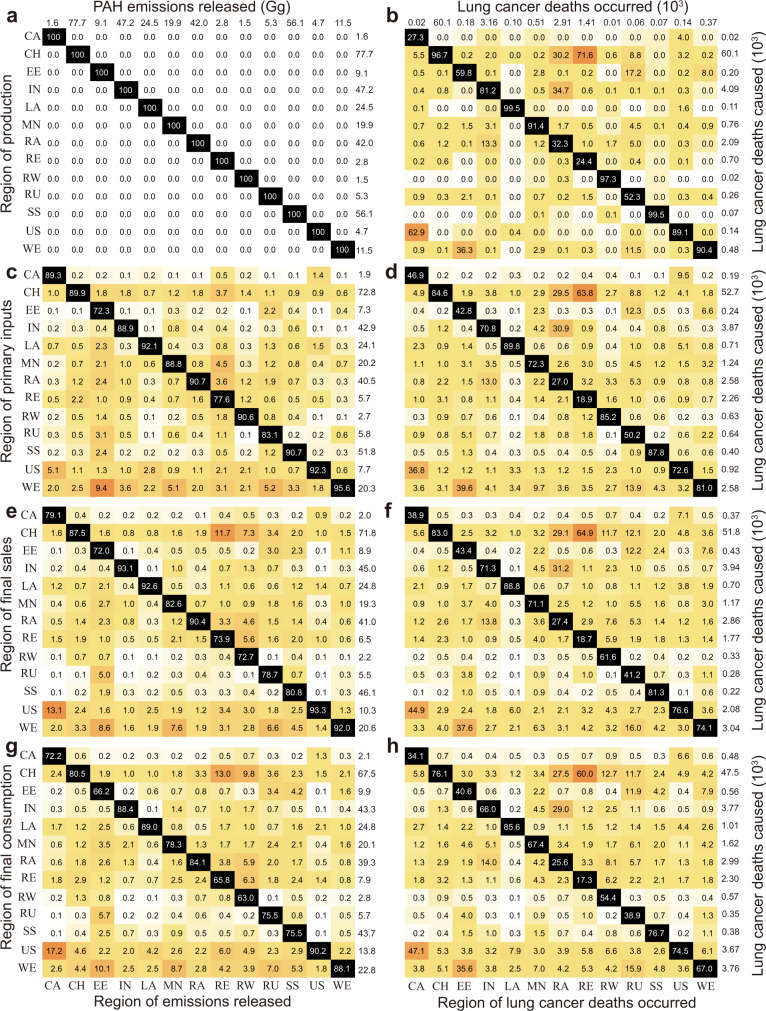
Composition of PAH emissions and related health impacts in 13 regions. The number and percentage of PAH emissions and PAH-related lifetime lung cancer deaths occurred in each region attributed to each region’s production (**a**, **b**), primary inputs (**c**, **d**), final sales (**e**, **f**), and final consumption (**g**, **h**) in 2015. Each cell in the grid shows the fraction of indicator (%) that occurred in the column region caused by production, primary input, final sales, and final consumption of the row regions. The total emitted emissions and occurred deaths are shown at the top of **a** and **b**. The diagonal black-colored cell represents the fraction of indicators caused by local activities. Darker orange cells refer to higher fractions.

Figure 2 shows the number and percentage of emissions and deaths that occurred in each region due to the production, primary inputs, final sales, and final consumption of all regions. From the perspective of pollution indicators, the influence of international trade on driving the PAH-related health risks was stronger than that on driving the emissions. For most regions, the percentage of local deaths attributed to external primary inputs, final sales, and final consumption was larger than that of emissions. This is because the transfer of emissions was related to the changes in manufacturing locations driven by international trade, while the transfer of health impacts was attributed to the combined effects of transboundary atmospheric transport and international trade. For instance, 34% of deaths in India were attributed to other regions’ consumption, whereas the percentage of released emissions in India related to external consumption was only 11.6% (Fig. 2g, h). The results indicate that more attention should be paid to the more serious transboundary health impacts of PAH pollution.

The transboundary health impacts related to international trade were larger than those associated with physical atmospheric transport of production emissions. In general, although local emissions contributed to higher health impacts in most regions (Fig. 2b), the health risks of the neighboring regions can be strongly affected by physical atmospheric transport. For instance, 34.7% and 30.2% of lifetime lung cancer deaths that occurred in the rest of Asia were caused by the production-based emissions of India and China through atmospheric transport, respectively (Fig. 2b). However, international trade could influence the health impacts between distant regions. For example, the emissions produced in western Europe and the USA almost had no impact on the health risks in China, whereas 5.3% (3.2 × 10^3^ deaths) and 5.1% (3.1 × 10^3^ deaths) of lifetime lung cancer deaths in China were attributed to the consumption in western Europe and the USA, respectively (Fig. 2h). Globally, the lifetime lung cancer deaths attributed to other regions’ consumption (27.9%, 1.9 × 10^4^ deaths) were larger than those related to other regions’ production (8.4%, 5.8 × 10^3^ deaths).

From the perspective of the economic supply chain, worldwide regions had various characteristics of the influence on health impacts in other regions. For instance, the contributions of primary inputs of Russia to other regions’ health impacts were larger than those related to their final sales and consumption (Supplementary Fig. 3). These regions are in the upstream stages of the global supply chain and mainly serve as resource suppliers of other regions, explaining the higher influences of their primary inputs on the health impacts in other regions. In addition, due to the large export of products, the lifetime lung cancer deaths that occurred in other regions caused by the final sales of China and India were larger than those attributed to their primary inputs and final consumption (Supplementary Fig. 3). For most regions, final consumption plays a more important role in the transfer of emissions and health risks than primary inputs and final sales. For instance, the consumption of the USA caused 3.6 × 10^3^ deaths in other regions, whereas its primary inputs and final sales resulted in 8.2 × 10^2^ and 2.0 × 10^3^ deaths in other regions (Fig. 2d, f, h). Considering the overall influences of three perspectives, the global PAH emissions (18.4%) and PAH-related deaths (27.9%) attributed to other regions’ consumption were larger than those related to other regions’ primary inputs (10.4%, 20.1%), and final sales (13.0%, 21.4%). The results entail that the optimization of final consumption patterns may be more effective in the mitigation of the PAH emissions and related health impacts.

### Net flow patterns of emissions and health impacts

**Fig. 3:**
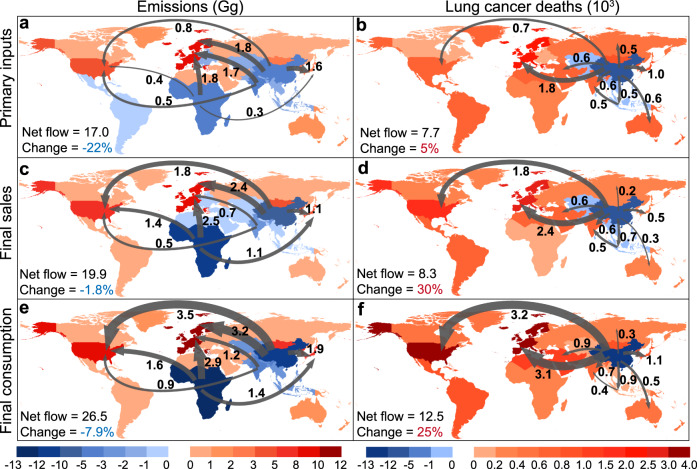
Global net flow patterns of PAH emissions and associated health impacts. The net flow patterns of income-based (**a**, **b**), final sale-based (**c**, **d**), and consumption-based (**e**, **f**) PAH emissions and lifetime lung cancer deaths in 13 regions in 2015. Red shades represent the net importers, while blue shades represent the net exporters. The arrows represent the net exported emissions or health losses from one region to another region, and the size of the arrow represents the level of net effect. The total net flow in 2015 and the percentage of its change from 2012 to 2015 are shown in the bottom-left corner.

The narrowing net flow patterns were observed between PAH emissions and health impacts. Figure 3 shows the net flow patterns of PAH emissions and health impacts in 13 regions in 2015 from the perspective of the global supply chain. When the emissions and health impacts occurred in other regions attributed to one region’s economic activities (including primary input, final sales, and final consumption) were larger than those occurring in this region attributed to other regions’ economic activities, it can be regarded as a net importer, otherwise, it is a net exporter. Results show that most of the developed regions were the net importers of emissions and health impacts. For instance, the total net inflow attributed to the overall consumption of the USA and western Europe accounted for 77% (20.5 Gg) and 55% (6.9 × 10^3^ deaths) of the total net inflow of global emissions and deaths, respectively. Furthermore, the range of net exporter regions was narrowing from the perspective of emissions to health impacts. For PAH emissions, China, the rest of Asia, and sub-Saharan Africa were the main net exporters from three perspectives of the supply chain due to their roles as producers in the global supply chain (Fig. 3a, c, e). For lifetime lung cancer deaths, China was the only net exporter (1.25 × 10^4^ deaths) from a consumption perspective (Fig. 3f), which is attributed to the large percentage of export, high PAH emission intensity, dense population, and high lung cancer rates.

Larger net flows of PAH emissions and health impacts occurred at the downstream of the economic supply chain. For example, the net flows induced by final consumption (26.5 Gg) were larger than those induced by primary inputs (17.0 Gg) and final sales (19.9 Gg) (Fig. 3a, c, e). Explicitly, the consumption-based net flow of lifetime lung cancer deaths (3.2 × 10^3^ deaths) from China to the USA was approximately five and two times that attributed to the primary inputs (0.7 × 10^3^ deaths) and final sales (1.8 × 10^3^ deaths), respectively (Fig. 3b, d, f). The results indicate that as the last stage of the economic supply chain, the final consumption had a greater influence on the net effect of PAH emissions and health impacts.

From 2012 to 2015, opposite trends of net flow amount were determined for emissions and health impacts. The net flow patterns of emissions and health impacts in 2012 and 2015 are provided in Supplementary Fig. 4 and Supplementary Table 4. Taking the example from the consumption perspective, the total net emission flow decreased by 7.9% from 2012 to 2015, but the total net flow of deaths increased by 25% (Fig. 3e, f). Although the net flow patterns for different years were similar, the trends in total net flow amount of emissions and health impacts were opposite, which might be related to the influences of socioeconomic determinates and environmental factors on these two indicators.

### Contributions of determinates to temporal changes

**Fig. 4:**
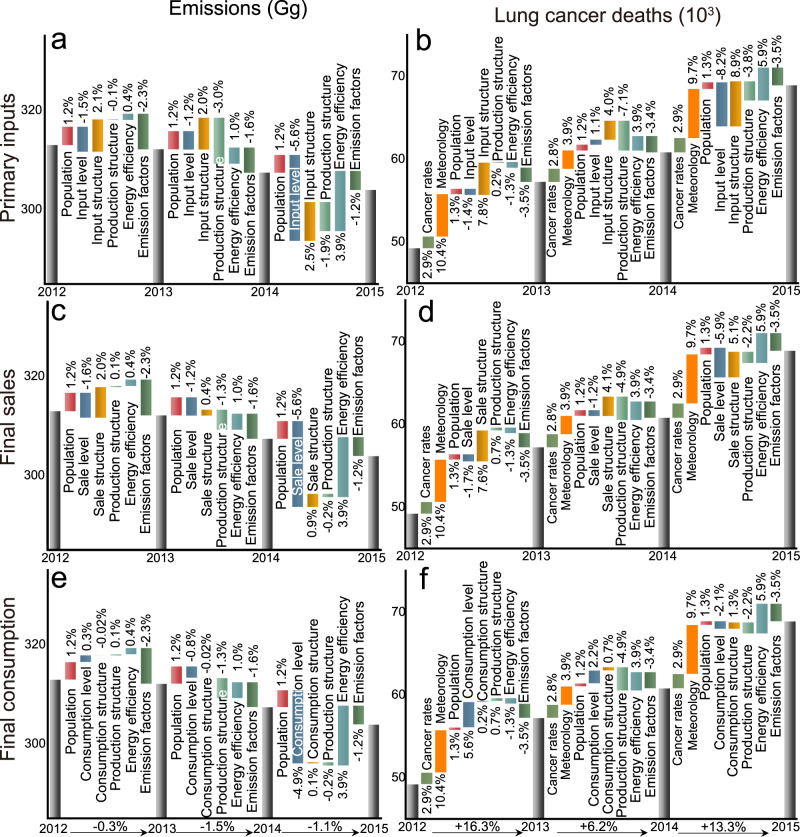
Drivers of global PAH emissions and associated health impacts from 2012 to 2015. The contributions of different drivers to the changes in global PAH emissions and lifetime lung cancer deaths from 2012 to 2015 from the perspectives of primary inputs (**a**, **b**), final sales (**c**, **d**), and final consumption (**e**, **f**). Noted that the production structure for primary inputs represents the production output structure (Ghosh inverse matrix), while that for final sales and final consumption represents the production input structure (Leontief inverse matrix).

The global emissions slightly decreased by 2.8% from 2012 to 2015, while the total lifetime lung cancer deaths increased by 40.3%. Figure 4 shows the contributions of socioeconomic determinates and environmental factors to the changes in the global PAH emissions and lifetime lung cancer deaths from 2012 to 2015 from the perspectives of primary input, final sale, and final consumption.

By combining the SDA, chemical transport model, and risk assessment, meteorological change was identified as one of the most important factors driving the increase in health impacts. For example, the meteorological changes enabled the global lifetime lung cancer deaths to increase by 10.4% from 2012 to 2013 and by 9.7% from 2014 to 2015 (Fig. 4d). Among the meteorological factors, temperature and particulate matter might be the two main factors driving the increase in PAH concentrations. Global temperature and particulate matter were found to increase during this period^27,28^. It is reported that the higher temperature made the PAH air-surface fluxes become net volatilization, indicating that more PAHs were presented in the atmosphere^26^. Then, since heavy PAHs (such as B*a*P) tend to be particle-bound, the higher concentration of particulate matter adsorbed more atmospheric PAHs, resulting in higher PAH concentrations^29^. Thus, the decrease in health impacts of PAH caused by reducing emissions through socioeconomic determinates might be offset by the influences of meteorological changes.

From the perspective of supply chains, mitigation policies should be focused on different aspects. On one hand, there is a great potential to mitigate PAH-related health impacts by reducing the final consumption level. For instance, the decrease in lifetime lung cancer deaths caused by the final consumption level was 2.1% from 2014 to 2015, which was lower than those caused by the primary input (8.2%) and final sale levels (5.9%) (Fig. 4b, d, f). Furthermore, the overall effect of final consumption level on health impacts from 2012 to 2015 was even driving the increase in health impacts (2.7 × 10^3^ deaths), while those of primary input and final sale levels led to the decrease in deaths (Supplementary Data 3, 4). On the other hand, the contributions of the primary input structure (8.9%) and final sale structures (5.1%) to the increase in the health impacts from 2014 to 2015 were larger than those of the final consumption structure (1.3%), entailing that more attention should be paid to the optimization of primary input and final sale structures (Fig. 4b, d, f). Therefore, it is essential to mitigate the PAH emissions and health impacts from the whole economic supply chain, such as reducing the final consumption levels and optimizing the primary input and final sale structures.

In terms of temporal changes, the impacts of socioeconomic determinates increased in the recent period. On one hand, the benefits of the changes in the supply chain have occurred in recent years. For instance, the influence of primary input level on decreasing the emissions increased from 1.5% to 5.6% from 2012 to 2015 (Fig. 4a). On the other hand, declines in energy efficiency became one of the most important drivers of increasing the emissions and health impacts among socioeconomic determinates, whose contributions to deaths changed from −1.3% to 5.9% over time (Fig. 4f). In addition, due to the development of science and technology, the declining emission factors led to decreases in the emissions and health impacts.

**Fig. 5:**
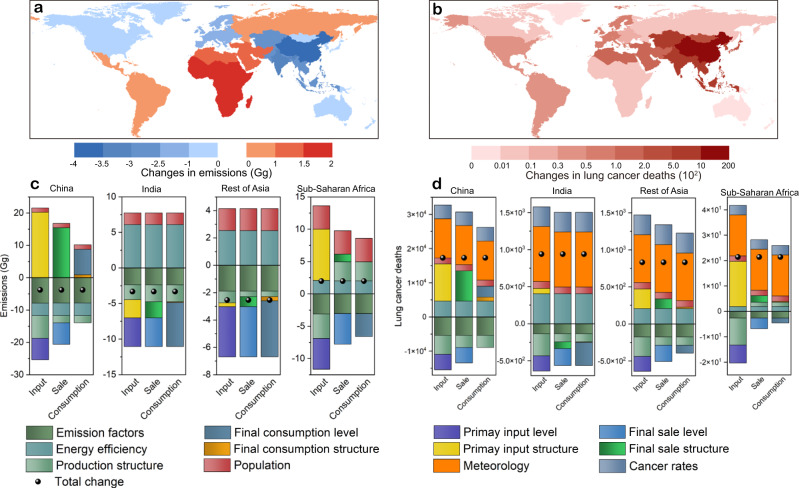
Contributions of drivers to changes in regional PAH emissions and related health impacts. **a**, **b** The changes in PAH emissions and PAH-related lifetime lung cancer deaths that occurred in 13 regions from 2012 to 2015; **c**, **d** the contributions of different drivers to the changes in emissions and deaths from the perspectives of primary inputs, final sales and final consumption for several regions (China, India, Rest of Asia, and Sub-Saharan Africa).

From the regional perspective, Fig. 5 shows the changes in the emissions and lifetime lung cancer deaths that occurred in 13 regions and the contributions of different drivers to the changes from 2012 to 2015 in several regions. For the temporal changes in emissions, China had the largest decrease (3.8 Gg) from 2012 to 2015 mainly driven by the emission factors, while Sub-Saharan Africa had the largest increase (2.0 Gg) mainly driven by the population (Fig. 5a). For the temporal changes in health impacts, the largest increase (1.7 × 10^4^ deaths) occurred in China, followed by that in India (9.4 × 10^2^ deaths) and the rest of Asia (8.4 × 10^2^ deaths) (Fig. 5b). Although the factor driving the increase in most regions was the meteorological change, international trade had a strong influence on the health impacts. For example, the primary input structure, final sale structure, and final consumption level accounted for 66%, 55%, and 24% of the net increase in Chinese health impacts, while the declines in energy efficiency contributed 41% of the net increase in lung cancer deaths in India (Fig. 5b). Thus, future global mitigation requires the optimization of the economic supply chain and the improvement of energy efficiency. The details about regional changes and driving factors are provided in Supplementary Note 4 and Supplementary Figs. 5–7.

## Discussion

This study investigated the influences of international trade on PAH emissions and lifetime lung cancer deaths from the different stages of the global supply chain. The income-, production-, final sale- and final consumption-based accounting methods allocated the responsibilities of emissions and health impacts to the primary suppliers, producers, final sellers, and final consumers, respectively. Thus, this study provides scientific suggestions and measures for mitigating global PAH emissions and related health risks from different aspects of economic supply chains.

The production-based accounting highlights the regions with large direct emissions, thus reducing emission factors and improving energy efficiency in these regions can assist in the mitigation of global PAH emissions. As the major emitters and exporters of emissions and health risks, China, India, and the rest of Asia have put more effort into improving the emission control technologies to reduce the emission factors. For example, the decreasing emission factors led to the reduction of PAH emissions (7.8 Gg) and health impacts (5.4 × 10^3^ deaths) in China from 2012 to 2015 (Fig. 5). However, the emission factors in these developing regions were still higher than those in developed regions (Supplementary Table 2). Thus, transferring advanced PAH abatement technologies from developed regions to developing regions is a promising way to reduce global PAH emissions in the future. In addition, more energy was consumed in high-PAH-pollution sectors to generate one-unit total output in India and the rest of Asia, leading to the declines in energy efficiency and related increase in PAH emissions (India: 6.1 Gg, the rest of Asia: 2.5 Gg) (Fig. 5). Thus, improving energy efficiency could be an effective mitigation strategy in these regions.

As the major net importers of PAH emissions, developed regions (western Europe, the USA, and the rest of east Asia) should focus on the mitigation strategies from the income-, final sale-, and final consumption-based perspectives. The net flows of PAH emissions and health impacts were mainly from developing regions to developed regions, due to their different roles in the global supply chain (Fig. 3). Explicitly, the exported PAH-related health losses in China were the largest (income:7.3 × 10^3^, sale: 8.3 × 10^3^, consumption: 1.3 × 10^4^), and most of them were related to developed regions’ primary input, final sale, and final consumption.

From the income-based perspective, limiting the loads and subsiding the investment of high-income-based sectors (e.g., petroleum and coal mining), and providing financial incentives for selling feedstocks to low-emission users should be considered^30^. Furthermore, transferring related technologies and capital investment to their downstream users could assist the global PAH mitigation. Moreover, since primary input structure played an important role in the changes in PAH emissions (Fig. 4), improving investment behaviors through investing and cultivating emerging industrials oriented by cleaner energy could achieve social-economic-natural sustainable development.

From the final sale-based perspective, identifying critical final sellers (e.g., construction and commercial services, Supplementary Fig. 8) can provide additional suggestions for policies focusing on production efficiency improvement^31–33^. Improving productivity means using fewer inputs of products (especially PAH-intensive inputs) from upstream providers to fulfill the demand of final consumers, which can reduce the upstream emissions^34^. According to the SDA results (Fig. 4), the final sale structure was one of the factors driving the increase in emissions and health impacts. To optimize the final sale structure, governments can tax intermediate goods based on embodied emissions (Supplementary Fig. 8), which will encourage key final sellers to improve their productivity and choose products with fewer upstream emissions^21^.

From the consumption-based perspective, since the final consumption level was identified as the factor driving the global PAH-related health impacts (Fig. 4), it is essential to affect consumption behaviors by placing environmental labels and changing consumption taxes. The environmental labels on products represent that the products meet the demand for environmental protection, and fewer emissions are emitted within the life cycle of the products. Thus, the environmental labels can promote PAH mitigation by assisting consumers to distinguish environmentally friendly products. Furthermore, lowering tax rates on these products and providing subsidies to their manufacturers can be applied to support this policy and further improve consumption behaviors.

Compared with other stages of the supply chain, final consumption transferred more PAH emissions and health impacts than primary inputs and final sales. The global PAH emissions attributed to other regions’ consumption (56 Gg) were larger than those related to other regions’ primary inputs (32 Gg) and final sales (39 Gg). Due to the high population density, high lung cancer rates, and large exports, China had the largest PAH-related lifetime lung cancer deaths (6.0 × 10^4^ deaths), and 24% of them were attributed to other regions’ consumption (Fig. 2). Thus, compared to other mitigation strategies, improving consumption behaviors may be more effective in reducing the global PAH-related health impacts. Furthermore, the mitigation strategies should be focused on the key sectors of PAH pollution (petroleum products, metal products, transport, commercial services, and private households) (Supplementary Notes 5, 6, Supplementary Fig. 8). It is essential to have the awareness that the investment, production, sales, and consumption of pollution-intensive products can eventually cause poor air quality and related lives lost in the world no matter in the local region or other regions. In general, global cooperation is the most efficient way to reduce the global PAH-related health impacts, such as applying different mitigation strategies at different stages of the supply chain.

International trade had been changed by several emerging factors, which also affected the flow patterns of PAH emissions and related health impacts. For instance, it is reported that the rise of South-South trade has transferred some of the export capacities from China to other emerging markets^35^. The ongoing Sino-US trade restrictions might reduce the global health impacts induced by international trade^36^. However, the COVID-19 crisis disrupted global supply chains, resulting in more exports from developing regions. Under the complex international situation, global collaborative actions become more important for supporting such pollution mitigation efforts and reducing the PAH-related health impacts.

## Methods and materials

### An integrated framework

In this study, an integrated approach is applied to estimate the global PAH emissions and lifetime lung cancer deaths from the perspective of the whole supply chain in the international trades (primary inputs, production, final sales, final consumption) and to determine the contributions of different drivers to the changes of two indicators. The primary input is the investment in sectors in all regions, supporting the production process. Final sale refers to the total products sold from a certain sector in a region to meet the consumption of all regions. Final consumption represents the final demand of a certain region for all sectors’ products. A detailed explanation of different accountings has been provided in Supplementary Note 3 and Supplementary Fig. 1. The overall framework is shown in Supplementary Fig. 2.

First, the global PAH emission inventories were generated in this study based on the data of emission factors, fuel consumption, and industrial processes.

Second, the environmentally extended multi-regional input-output (EE-MRIO) model was employed to reallocate the responsibilities of emissions based on each region’s primary inputs, final sales, and final consumption. Within the model, the emission inventories were linked with the MRIO table through emission intensity. Then, due to the 13 aggregated worldwide regions and four supply chain perspectives, there are 52 (4 × 13) scenario-specific emission maps for each year. The details of all scenarios are presented in Supplementary Data 5.

Third, the GEOS-Chem model was applied to derive the surface B*a*P concentrations of each scenario by simulating the physical transport of B*a*P emissions in the atmosphere. Since B*a*P is the most toxic pollutant among the PAHs, its simulation could represent the health impacts of PAHs^37^. Through inputting the scenario-specific emission obtained from the MRIO model, the scenario-specific concentrations can be simulated.

Fourth, based on the simulated concentrations and the Global Burden of Disease (GBD) database, the lifetime lung cancer risk assessment was used to evaluate the PAH-related lung cancer deaths in each scenario.

Finally, the structural decomposition analysis (SDA) was employed to determine the contributions of socioeconomic determinates to the changes in PAH emissions from 2012 to 2015. Then, the health impacts driven by socioeconomic determinates and other factors (meteorological change, lung cancer rates) were obtained by combing the results of SDA, GEOS-Chem model, and lifetime lung cancer risk assessment. The uncertainty of the integrated model has been estimated by Monte Carlo simulation (Supplementary Fig. 9)^20,38,39^, and the details about the uncertainty analysis and limitations are provided in Supplementary Notes 7, 8.

### PAH emission inventory

The global PAH emission inventories with different sectors in different regions from 2012 to 2015 were generated in this study. Sixteen priority PAHs were included in the inventory, which are naphthalene (NAP), acenaphthene (ACE), acenaphthylene (ACY), fluorene (FLU), anthracene (ANT), phenanthrene (PHE), fluoranthene (FLUH), pyrene (PYR), benzo[*a*]anthracene (B*a*A), chrysene (CHR), benzo[*b*]fluoranthene (B*b*F), benzo[*k*]fluoranthene (B*k*F), benzo[*a*]pyrene (B*a*P), dibenzo[*a,h*]anthracene (DBA), indeno[*1,2,3-cd*]pyrene (IDP), and benzo[*g,h,i*]perylene (B*g*P). The production-based global emission inventory of PAHs was generated by multiplying fuel consumption and activity rates with different emission factors in various sectors in different countries:

1 $${{{{\boldsymbol{E}}}}}_{{{{\boldsymbol{i}}}},{{{\boldsymbol{m}}}}}=\mathop{\sum}\limits_{{{{\boldsymbol{k}}}}}{{{{\boldsymbol{FC}}}}}_{{{{\boldsymbol{i}}}},{{{\boldsymbol{m}}}}}^{{{{\boldsymbol{k}}}}}\times {{{{\boldsymbol{EF}}}}}_{{{{\boldsymbol{i}}}},{{{\boldsymbol{m}}}}}^{{{{\boldsymbol{k}}}}}+\mathop{\sum}\limits_{{{{\boldsymbol{p}}}}}{{{{\boldsymbol{AR}}}}}_{{{{\boldsymbol{i}}}},{{{\boldsymbol{m}}}}}^{{{{\boldsymbol{p}}}}}\times {{{{\boldsymbol{EF}}}}}_{{{{\boldsymbol{i}}}},{{{\boldsymbol{m}}}}}^{{{{\boldsymbol{p}}}}}$$

where *E*_*i,m*_ indicates PAH emissions released from consumed energy type *k* (including coal, natural gas, petroleum, and biomass) and industrial processes in sector *i* in country *m*, *FC* indicates the fuel consumption of different energy types, *AR* indicates the activity rate, representing produced products in different industrial process *p*, *EF* indicates the emission factor.

The global fuel consumption was obtained from the International Energy Agency (IEA), which includes 69 energy types in 103 flows (28 sectors) in 186 worldwide regions. The industrial processes contain primary aluminum production, pig iron production, crude steel production, coke production, petroleum catalytic cracking, and agricultural residue burning. The activity rates of these processes in all countries were derived from different data sources (Supplementary Data 1). The emission factors of PAHs for 69 sources were obtained from literature^13^. In addition, emission factors varied between countries due to the difference in technologies. Through the technology splitting approach, the source-specific emission factors were distinguished based on different emission mitigation measures in countries. The details about emission factors and the comparison with previous PAH inventories are presented in Supplementary Notes 1, 2 (Supplementary Data 2).

### Income, final sale, and consumption-based accounting

The EE-MRIO model has been widely applied in analyzing the influences of increasing international trade on pollution^21,40,41^. In this study, after obtaining the production-based emissions, the EE-MRIO model was employed to estimate the region-specific income-, final sale-, and consumption-based PAH emissions. The different emission accounting represents PAH pollutants produced throughout the supply chain caused by certain primary inputs, final sales, and final consumption. The EE-MRIO model can track PAH emissions from the region of primary input, final sales, and final consumption to the region of production through international trade. In the EE-MRIO framework, based on the detailed trade data about the import and export among all sectors of regions, all produced PAH emissions can be assigned to the primary suppliers, final sellers, and final consumers. In other words, a certain region can take the responsibility for PAH emissions emitted from the production of the goods and services due to this region’s primary input, final sale, and final consumption.

The latest MRIO tables for 2012, 2013, 2014, and 2015 were obtained from Eora (www.worldmrio.com, accessed Feb. 2021)^42^, which contains 190 regions and 26 sectors (Supplementary Data 6). Due to their time continuity, large coverage of countries and sectors, and sufficient data of final demand and primary input, the Eora MRIO tables were chosen. To make the temporal results comparable, the tables were converted into 2015 prices with a double deflation method based on the GDP deflator of different regions obtained from the World Bank (www.data.worldbank.org, assessed Feb. 2021)^43^. In addition, the MRIO tables and PAH emissions have been aggregated into 17 sectors to match each other (Supplementary Table 5). Furthermore, since deforestation and wildfire are not related to economic activities, the PAH emissions from natural sources were excluded from the MRIO model.

Assuming that there are *m* regions with *i* sectors in each region (superscripts and subscripts *m* and *n* are region identifiers; *i* and *j* are sector identifiers), the basic linear equation of the input-output model is:

2 $${{{\boldsymbol{X}}}}={({{{\boldsymbol{I}}}}-{{{\boldsymbol{A}}}})}^{-{{{\bf{1}}}}}{{{\boldsymbol{F}}}}$$

3 $$X={V(I-B)}^{-1}$$

*X* represents the total output, whose element ${x}_{i}^{m}$ indicates the sum of intermediate use in all sectors and the final demand of *n* regions for sector *i* in region *m*. *I* represents the identity matrix. The direct input coefficient matrix *A* can be expressed as:

4 $${{{\boldsymbol{A}}}}=\left[\begin{array}{c}\begin{array}{c}{{{{\boldsymbol{A}}}}}^{{{{\bf{11}}}}}\\ {{{{\boldsymbol{A}}}}}^{{{{\bf{21}}}}}\end{array}\\ \vdots \\ {{{{\boldsymbol{A}}}}}^{{{{\boldsymbol{m}}}}{{{\bf{1}}}}}\end{array}\begin{array}{ccc}\begin{array}{c}{{{{\boldsymbol{A}}}}}^{{{{\bf{12}}}}}\\ {{{{\boldsymbol{A}}}}}^{{{{\bf{22}}}}}\end{array} & \begin{array}{c}\cdots \\ \cdots \end{array} & \begin{array}{c}{{{{\boldsymbol{A}}}}}^{{{{\bf{1}}}}{{{\boldsymbol{n}}}}}\\ {{{{\boldsymbol{A}}}}}^{{{{\bf{2}}}}{{{\boldsymbol{n}}}}}\end{array}\\ \vdots & \ddots & \vdots \\ {{{{\boldsymbol{A}}}}}^{{{{\boldsymbol{m}}}}{{{\bf{2}}}}} & \cdots & {{{{\boldsymbol{A}}}}}^{{{{\boldsymbol{mn}}}}}\end{array}\right]$$

5 $${A}^{{mn}}=\left[\begin{array}{c}\begin{array}{c}{a}_{11}^{{mn}}\\ {a}_{21}^{{mn}}\end{array}\\ \vdots \\ {a}_{i1}^{{mn}}\end{array}\begin{array}{ccc}\begin{array}{c}{a}_{12}^{{mn}}\\ {a}_{22}^{{mn}}\end{array} & \begin{array}{c}\cdots \\ \cdots \end{array} & \begin{array}{c}{a}_{1j}^{{mn}}\\ {a}_{2j}^{{mn}}\end{array}\\ \vdots & \ddots & \vdots \\ {a}_{i2}^{{mn}} & \cdots & {a}_{{ij}}^{{mn}}\end{array}\right]$$

6 $${a}_{{ij}}^{{mn}}=\frac{{z}_{{ij}}^{{mn}}}{{x}_{j}^{n}}$$

The direct output coefficient matrix *B* can be expressed as:

7 $${{{\boldsymbol{B}}}}=\left[\begin{array}{c}\begin{array}{c}{{{{\boldsymbol{B}}}}}^{{{{\bf{11}}}}}\\ {{{{\boldsymbol{B}}}}}^{{{{\bf{21}}}}}\end{array}\\ \vdots \\ {{{{\boldsymbol{B}}}}}^{{{{\boldsymbol{m}}}}{{{\bf{1}}}}}\end{array}\begin{array}{ccc}\begin{array}{c}{{{{\boldsymbol{B}}}}}^{{{{\bf{12}}}}}\\ {{{{\boldsymbol{B}}}}}^{{{{\bf{22}}}}}\end{array} & \begin{array}{c}\cdots \\ \cdots \end{array} & \begin{array}{c}{{{{\boldsymbol{B}}}}}^{{{{\bf{1}}}}{{{\boldsymbol{m}}}}}\\ {{{{\boldsymbol{B}}}}}^{{{{\bf{2}}}}{{{\boldsymbol{m}}}}}\end{array}\\ \vdots & \ddots & \vdots \\ {{{{\boldsymbol{B}}}}}^{{{{\boldsymbol{m}}}}{{{\bf{2}}}}} & \cdots & {{{{\boldsymbol{B}}}}}^{{{{\boldsymbol{mm}}}}}\end{array}\right]$$

8 $${B}^{{mn}}=\left[\begin{array}{c}\begin{array}{c}{b}_{11}^{{mn}}\\ {b}_{21}^{{mn}}\end{array}\\ \vdots \\ {b}_{i1}^{{mn}}\end{array}\begin{array}{ccc}\begin{array}{c}{b}_{12}^{{mn}}\\ {b}_{22}^{{mn}}\end{array} & \begin{array}{c}\cdots \\ \cdots \end{array} & \begin{array}{c}{b}_{1j}^{{mn}}\\ {b}_{2j}^{{mn}}\end{array}\\ \vdots & \ddots & \vdots \\ {b}_{i2}^{{mn}} & \cdots & {b}_{{ij}}^{{mn}}\end{array}\right]$$

9 $${b}_{{ij}}^{{mn}}=\frac{{z}_{{ij}}^{{mn}}}{{x}_{i}^{m}}$$

where ${a}_{{ij}}^{{mn}}$ indicates the direct consumption coefficient representing the direct intermediate use from sector *i* in region *m* to generate one monetary unit of production of sector *j* in the region *n*; ${b}_{{ij}}^{{mn}}$ reflects the direct intermediate use from sector *j* in region *n* to generate one monetary unit of production of sector *i* in the region *m;* ${z}_{{ij}}^{{mn}}$ indicates the intermediate monetary flows from sector *i* in region *m* to sector *j* in region *n*. (*I-A*)^−1^ indicates the Leontief inverse matrix, and (*I-B*)^−1^ indicates the Ghosh inverse matrix.

10 $${{{\boldsymbol{F}}}}=\left[\begin{array}{c}\begin{array}{c}{{{{\boldsymbol{f}}}}}_{{{{\bf{1}}}}}^{{{{\bf{11}}}}}\\ {{{{\boldsymbol{f}}}}}_{{{{\bf{2}}}}}^{{{{\bf{11}}}}}\end{array}\\ \begin{array}{c}\vdots \\ {{{{\boldsymbol{f}}}}}_{{{{\boldsymbol{i}}}}}^{{{{\bf{11}}}}}\\ \vdots \end{array}\\ {{{{\boldsymbol{f}}}}}_{{{{\boldsymbol{i}}}}}^{{{{\boldsymbol{m}}}}{{{\bf{1}}}}}\end{array}\begin{array}{ccc}\begin{array}{c}{{{{\boldsymbol{f}}}}}_{{{{\bf{1}}}}}^{{{{\bf{12}}}}}\\ {{{{\boldsymbol{f}}}}}_{{{{\bf{2}}}}}^{{{{\bf{12}}}}}\end{array} & \begin{array}{c}\cdots \\ \cdots \end{array} & \begin{array}{c}{{{{\boldsymbol{f}}}}}_{{{{\bf{1}}}}}^{{{{\bf{1}}}}{{{\boldsymbol{n}}}}}\\ {{{{\boldsymbol{f}}}}}_{{{{\bf{2}}}}}^{{{{\bf{1}}}}{{{\boldsymbol{n}}}}}\end{array}\\ \begin{array}{c}\vdots \\ {{{{\boldsymbol{f}}}}}_{{{{\boldsymbol{i}}}}}^{{{{\bf{12}}}}}\\ \vdots \end{array} & \ddots & \begin{array}{c}\vdots \\ {{{{\boldsymbol{f}}}}}_{{{{\boldsymbol{i}}}}}^{{{{\bf{1}}}}{{{\boldsymbol{n}}}}}\\ \vdots \end{array}\\ {{{{\boldsymbol{f}}}}}_{{{{\boldsymbol{i}}}}}^{{{{\boldsymbol{mn}}}}} & \cdots & {{{{\boldsymbol{f}}}}}_{{{{\boldsymbol{i}}}}}^{{{{\boldsymbol{mn}}}}}\end{array}\right]$$

11 $${{{\rm{y}}}}=\left[\begin{array}{c}\begin{array}{c}\mathop{\sum}\limits_{n}{f}_{1}^{1n}\\ \vdots \end{array}\\ \mathop{\sum}\limits_{n}{f}_{i}^{1n}\\ \begin{array}{c}\vdots \\ \mathop{\sum}\limits_{n}{f}_{i}^{{mn}}\end{array}\end{array}\right]$$

12 $${{{\rm{v}}}}=\left[\begin{array}{ccc}{v}_{1}^{1} & \cdots & \begin{array}{ccc}{v}_{j}^{1} & \cdots & {v}_{j}^{n}\end{array}\end{array}\right]$$

where *F* indicates the matrix of final demand, whose element ${f}_{i}^{{mn}}$ represents the total final demand in region *n* for the products of sector *i* in region *m*; *y* indicates the vector of final sales, whose element $\mathop{\sum}\limits_{n}{f}_{i}^{{mn}}$ refers to the sum of total products sold from sector *i* in region *m* to all destination regions; *v* indicates the vector of primary input, whose element ${v}_{j}^{n}$ represents the primary input of sector *j* in region *n*.

To link PAH emissions with the monetary flow, the emission intensity *u*, indicating the ratio of the total emissions of sector *i* to the total output of sector *i*, can be expressed as follows:

13 $${{{\boldsymbol{u}}}}=\left[\begin{array}{c}\begin{array}{c}{{{{\boldsymbol{u}}}}}^{{{{\bf{1}}}}}\\ {{{{\boldsymbol{u}}}}}^{{{{\bf{2}}}}}\end{array}\\ \vdots \\ {{{{\boldsymbol{u}}}}}^{{{{\boldsymbol{m}}}}}\end{array}\right],\,{{{{\boldsymbol{u}}}}}^{{{{\boldsymbol{m}}}}}=\left[\begin{array}{c}\begin{array}{c}{{{{\boldsymbol{u}}}}}_{{{{\bf{1}}}}}^{{{{\boldsymbol{m}}}}}\\ {{{{\boldsymbol{u}}}}}_{{{{\bf{2}}}}}^{{{{\boldsymbol{m}}}}}\end{array}\\ \vdots \\ {{{{\boldsymbol{u}}}}}_{{{{\boldsymbol{i}}}}}^{{{{\boldsymbol{m}}}}}\end{array}\right],{{{{\boldsymbol{u}}}}}_{{{{\boldsymbol{i}}}}}^{{{{\boldsymbol{m}}}}}=\frac{{{{{\boldsymbol{E}}}}}_{{{{\boldsymbol{i}}}}}^{{{{\boldsymbol{m}}}}}}{{{{{\boldsymbol{X}}}}}_{{{{\boldsymbol{i}}}}}^{{{{\boldsymbol{m}}}}}}$$

where *u* represents a vector of the emission intensity for all sectors in all regions. Then, the PAH emissions attributed to primary input, final sale, and consumption can be expressed mathematically as:

14 $${{{{\boldsymbol{Q}}}}}_{{{{\boldsymbol{I}}}}}={{{\boldsymbol{V}}}}{({{{\boldsymbol{I}}}}-{{{\boldsymbol{B}}}})}^{-{{{\bf{1}}}}}{{{\boldsymbol{U}}}}$$

15 $${Q}_{S}=U{(I-A)}^{-1}Y$$

16 $${Q}_{C}=U{(I-A)}^{-1}F$$

Q_I_, Q_S_, Q_C_ indicate the total income-based, final sale-based, and consumption-based emission matrixes, whose element *Q*^*mn*^ represents the emissions released in region *m* for the primary input, final sale, and final consumption in region *n*. *U*, *V*, and *Y* are the diagonal matrix of *u, v*, and *y*.

Geographical aggregation was required due to the computational constraints and consumed time of each GEOS-Chem model run. According to the roles in international trade, pollution levels, and levels of economic development, the world was classified into 13 regions which is consistent with the previous research^3^: India, China, rest of east Asia, rest of Asia, Canada, USA, Latin America, eastern Europe, western Europe, Russia, Middle East and north Africa, Sub-Saharan Africa, and rest of the world (Supplementary Fig. 10). The result matrixes of Q_I_, Q_S_, Q_C_ were aggregated into these 13 regions so that the income-, production-, final sale-, and consumption-based emissions of 13 regions can be obtained.

### GEOS-Chem chemical transport model

GEOS-Chem is a global 3-D model of atmospheric chemistry driven by assimilated meteorological observations from the Goddard Earth Observing System (GEOS) of the NASA Global Modeling Assimilation Office (GMAO)^44^. This study employed the GEOS-Chem model (version 13.0.2) to simulate the global near-surface B*a*P concentrations of different emission scenarios.

The simulation for the persistent organic pollutants was chosen in the model run. This simulation can generate the concentrations of PAH in the gas phase, PAH partitioning in/onto organic carbon and black carbon aerosols through introducing temperature-dependent gas-particle partitioning^14^. Furthermore, the PAH loss through oxidation reaction with hydroxyl radical and O_3_ was considered in the simulation by using an empirically derived rate constant. In addition, wet depositions including rainout and washout from scavenging in convective updraft and precipitation for both gas and particulate PAH were compatible in the GEOS-Chem model by applying the temperature-dependent air-water partition coefficient and scavenging ratio. Dry deposition of PAH was included by calculating the dry deposition velocities with a resistance-in-series scheme. Moreover, the simulation was updated by calculating the soil-air and vegetation-air exchanges with a level-III fugacity model^29^. The incorporation of re-emissions of PAH increased the accuracy of the model results.

Before the running of the GEOS-Chem model, the input files need to be prepared. The income-, production-, final sale-, and consumption-based B*a*P emissions of 13 regions have been obtained through the construction of the PAH emission inventories and EE-MRIO model. The PAH emission distribution was based on 0.1° × 0.1°-resolution PKU-PAH inventory (www.inventory.pku.edu.cn, accessed Feb. 2021)^13^. Supposing that the spatial distribution patterns of the emissions did not change greatly, the B*a*P emission maps with different scenarios from 2012 to 2015 in this study were generated based on calculated country-specific income-based, production-based, final sale-based, and consumption-based emissions and PKU-PAH emission distributions.

Due to the high performance and convenience, the Amazon Web Services cloud computing platform was employed to run the GEOS-Chem model. In all runs, the simulations were conducted for the entire year with a 12-month spin-up in a 1-min time-frequency. The results of the simulation are 12 monthly average concentrations with 4° × 5° resolution and 47 vertical levels, which were further remapped into annual average concentrations with 1° × 1° resolution. The sum of B*a*P concentrations of the gas phase, particulate in/onto organic carbon, and black carbon aerosols at the bottom level of the model was considered as the ground-level concentrations (Supplementary Fig. 11).

The model validation was conducted by comparing the simulated B*a*P concentrations with the ground measurements from European Monitoring and Evaluation Programme (EMEP) (www.projects.nilu.no/ccc/emepdata, assessed Mar. 2021), and the National Air Pollution Surveillance Network of Canada (NAPS) (www.data.ec.gc.ca, assessed Mar. 2021), and observations in the world from literature (Supplementary Data 7). The locations of the observations from EMEP, NAPS, and previous studies are shown in Supplementary Fig. 12. The comparison between the simulated values and observation is presented in Supplementary Fig. 13. In general, the model-simulated concentrations agree well with the ground observations (*R*^2^ = 0.84), representing that the simulations are dependable.

### Lifetime lung cancer assessment

The lung cancer risk assessment based on the dose-response concept has been widely applied to estimate the lung cancer risks associated with the general exposure to ambient atmospheric PAH within a lifetime^9,45,46^. The population attributable fraction (PAF) is the percentage of lung cancer deaths caused by exposure to certain B*a*P concentrations in a lifetime. For each grid cell, PAF can be calculated as follows:

17 $${{{\bf{rr}}}}\left({{{{\bf{c}}}}}_{{{{\bf{BaP}}}}}\right)={\left[{{{{\bf{URR}}}}}_{{{{\bf{cum}}}}.{{{\mathbf{exp }}}}={{{\bf{100}}}}}\right]}^{({{{{\bf{c}}}}}_{{{{\bf{BaP}}}}}\times \frac{{{{\bf{70}}}}}{{{{\bf{100}}}}})}$$

18 $${{{\rm{PAF}}}}=\frac{{{{\rm{rr}}}}\left({{{{\rm{c}}}}}_{{{{\rm{BaP}}}}}\right)-1}{{{{\rm{rr}}}}\left({{{{\rm{c}}}}}_{{{{\rm{BaP}}}}}\right)}$$

where c_BaP_ indicates the near-surface BaP concentrations (μg∙m^−3^), URR is the unit relative risk and rr(c_BaP_) is the relative risk associated with a certain BaP concentration. URR_cum.exp=100_ refers to the unit relative risk at 100 μg∙m^−3^ years BaP, which is different between regions (Asia: 1.3, Europe: 1.13, north America: 1.16, the average value of the world: 1.2)^9^.

In this study, a lifetime exposure of 70 years was assumed, and the grids were at 1° × 1° resolution. The lifetime lung cancer deaths due to exposure to B*a*P in each grid cell can be calculated as:

19 $${{{{\bf{D}}}}}_{{{{\bf{BaP}}}}}={{{\bf{PAF}}}}\times \mathop{\sum }\limits_{{{{\bf{y}}}}={{{\bf{1}}}}}^{{{{\bf{70}}}}}{{{{\bf{LD}}}}}_{{{{\bf{y}}}},{{{\bf{m}}}}}$$

20 $${{{{\rm{LD}}}}}_{{{{\rm{y}}}},{{{\rm{m}}}}}={{{{\rm{LR}}}}}_{{{{\rm{y}}}},{{{\rm{m}}}}}\times {{{{\rm{P}}}}}_{{{{\rm{y}}}},{{{\rm{m}}}}}$$

21 $${{{{\rm{D}}}}}_{{{{\rm{BaP}}}},{{{\rm{m}}}}}={\sum }_{g}^{m}{{{{\rm{D}}}}}_{{{{\rm{BaP}}}}}$$

where D_BaP_ represents the B*a*P-related lung cancer deaths in the next 70 years in each grid cell, assuming people are exposed to certain B*a*P concentrations for the whole lifetime; D_BaP, m_ is the sum of D_BaP_ in all grid cells of the region *m*; $\mathop{\sum }\nolimits_{{{{\rm{y}}}}=1}^{70}{{{{\rm{LD}}}}}_{{{{\rm{y}}}},{{{\rm{m}}}}}$ refers to the sum of lung cancer deaths in countries *m* in the lifetime (70 years); LR_y,m_ and P_y,m_ are the lung cancer rate (deaths per 100,000 people) and population of certain country *m* in a certain year *y*. The lung cancer rates of countries were obtained from the Global Burden of Disease (GBD) database (www.healthdata.org, assessed Mar. 2021). The population for the next 70 years was obtained from World Population Prospects 2019 (www.population.un.org, assessed Mar. 2021)^47^. The population distributions for different years were obtained from the LandScan global population database (www.landscan.ornl.gov, assessed Mar. 2021) at a 1 km resolution, which was further aggregated to the same resolution of the B*a*P concentration (1° × 1°). The lifetime lung cancer deaths for base cases in each year can be estimated based on the above method, and those for each scenario can be calculated as follows. For example, a scenario is about the emissions due to the consumption of China in 2015. Firstly, the emissions caused by the consumption of China in 2015 were imported into the GEOS-Chem model to obtain the concentrations of this scenario. Then, the deaths attributed to the Chinese consumption-based emissions in each grid can be calculated by multiplying the base deaths and the fractional contribution of scenario concentration to base concentration^3^. Since the results cover all the grids, the deaths in each region caused by the Chinese consumption-based emissions can be obtained by summing the values in the region’s grid cells (Eq. 21). The sum of the scenario’s deaths in all grids is the Chinese consumption-based lifetime lung cancer deaths. Through the lifetime lung cancer assessment, the lifetime lung cancer deaths due to each region’s primary input, production, final sales, and final consumption can be estimated. The emissions and lifetime lung cancer deaths that occurred in each region from 2012 to 2015 are provided in Supplementary Table 6.

### Structural deposition analysis (SDA) for emissions

Input-output SDA has been widely employed for quantifying the influence of socioeconomic drivers on pollutant emissions^48,49^. To estimate the influence of drivers on the changes in PAH emissions in the world from 2012 to 2015, the income-, final sale-, and consumption-based emissions can be decomposed into six factors, as follows:

22 $${{{{\boldsymbol{Q}}}}}_{{{{\boldsymbol{I}}}}}={{{\boldsymbol{V}}}}\cdot {{{\boldsymbol{G}}}}\cdot {{{\boldsymbol{U}}}}={{{\boldsymbol{P}}}}\cdot {{{\boldsymbol{W}}}}\cdot {{{\boldsymbol{N}}}}\cdot {{{\boldsymbol{G}}}}\cdot {{{\boldsymbol{T}}}}\cdot {{{\boldsymbol{K}}}}$$

23 $${Q}_{S}=U \cdot L \cdot Y=K \cdot T \cdot L \cdot Z \cdot S \cdot P$$

24 $${Q}_{C}=U \cdot L \cdot F=K \cdot T \cdot L \cdot M \cdot R \cdot P$$

where *L* represents the Leontief inverse matrix indicating the production input structure, *G* represents the Ghosh inverse matrix indicating the production output structure, and *U* is the diagonal emission intensity matrix, which can be decomposed into two factors: *K* indicates the emission factors (PAH emissions per unit of energy consumption) and *T* indicates the energy efficiency (energy consumption per unit of total output and fuel mix of sectors); and *V*, *Y*, *F* are the primary input, final sale and final demand matrixes, which can be further decomposed into three explicit determinants: *N*, *Z*, and *M* are the primary input, final sale and final consumption structures (the share of each variable of total values); *W*, *S*, and *R* are the primary input, final sale and final consumption levels (variable per capita); and *P* is the population. Each of the six factors denotes the contributions to PAH emission change triggered by one driving factor when the other factors are kept constant. Theoretically, in this six-factor SDA model, the non-uniqueness problem will result in 6! types of first-order decomposition forms, and different results can be obtained from different procedures. Following previous studies^50,51^, two-polar decomposition using the arithmetic average of all possible first-order decomposition forms was applied to address this problem. Thus, the changes in consumption-based PAH emissions across different years can be expressed as follows:

25 $$\triangle {{{{\boldsymbol{Q}}}}}_{{{{\boldsymbol{C}}}}}=	\,\triangle {{{{\boldsymbol{Q}}}}}_{{{{\boldsymbol{K}}}}}+\triangle {{{{\boldsymbol{Q}}}}}_{{{{\boldsymbol{T}}}}}+\triangle {{{{\boldsymbol{Q}}}}}_{{{{\boldsymbol{L}}}}}+\triangle {{{{\boldsymbol{Q}}}}}_{{{{\boldsymbol{M}}}}}+\triangle {{{{\boldsymbol{Q}}}}}_{{{{\boldsymbol{R}}}}}+\triangle {{{{\boldsymbol{Q}}}}}_{{{{\boldsymbol{P}}}}}\\ =	\frac{\triangle {{{\boldsymbol{K}}}}{{{{{\boldsymbol{T}}}}}_{{{{\boldsymbol{t}}}}}{{{\boldsymbol{L}}}}}_{{{{\boldsymbol{t}}}}}{{{{\boldsymbol{M}}}}}_{{{{\boldsymbol{t}}}}}{{{{\boldsymbol{R}}}}}_{{{{\boldsymbol{t}}}}}{{{{\boldsymbol{P}}}}}_{{{{\boldsymbol{t}}}}}+\triangle {{{\boldsymbol{K}}}}{{{{{\boldsymbol{T}}}}}_{{{{\bf{0}}}}}{{{\boldsymbol{L}}}}}_{{{{\bf{0}}}}}{{{{\boldsymbol{M}}}}}_{{{{\bf{0}}}}}{{{{\boldsymbol{R}}}}}_{{{{\bf{0}}}}}{{{{\boldsymbol{P}}}}}_{{{{\bf{0}}}}}}{{{{\bf{2}}}}}\\ 	+\frac{{{{{\boldsymbol{K}}}}}_{{{{\bf{0}}}}}\triangle {{{\boldsymbol{T}}}}{{{{\boldsymbol{L}}}}}_{{{{\boldsymbol{t}}}}}{{{{\boldsymbol{M}}}}}_{{{{\boldsymbol{t}}}}}{{{{\boldsymbol{R}}}}}_{{{{\boldsymbol{t}}}}}{{{{\boldsymbol{P}}}}}_{{{{\boldsymbol{t}}}}}+{{{{\boldsymbol{K}}}}}_{{{{\boldsymbol{t}}}}}\triangle {{{\boldsymbol{T}}}}{{{{\boldsymbol{L}}}}}_{{{{\bf{0}}}}}{{{{\boldsymbol{M}}}}}_{{{{\bf{0}}}}}{{{{\boldsymbol{R}}}}}_{{{{\bf{0}}}}}{{{{\boldsymbol{P}}}}}_{{{{\bf{0}}}}}}{{{{\bf{2}}}}}\\ 	+\frac{{{{{\boldsymbol{K}}}}}_{{{{\bf{0}}}}}{{{{\boldsymbol{T}}}}}_{{{{\bf{0}}}}}{\triangle {{{\boldsymbol{LM}}}}}_{{{{\boldsymbol{t}}}}}{{{{\boldsymbol{R}}}}}_{{{{\boldsymbol{t}}}}}{{{{\boldsymbol{P}}}}}_{{{{\boldsymbol{t}}}}}+{{{{\boldsymbol{K}}}}}_{{{{\boldsymbol{t}}}}}{{{{\boldsymbol{T}}}}}_{{{{\boldsymbol{t}}}}}\triangle {{{\boldsymbol{L}}}}{{{{\boldsymbol{M}}}}}_{{{{\bf{0}}}}}{{{{\boldsymbol{R}}}}}_{{{{\bf{0}}}}}{{{{\boldsymbol{P}}}}}_{{{{\bf{0}}}}}}{{{{\bf{2}}}}}\\ 	+\frac{{{{{\boldsymbol{K}}}}}_{{{{\bf{0}}}}}{{{{\boldsymbol{T}}}}}_{{{{\bf{0}}}}}{{{{\boldsymbol{L}}}}}_{{{{\bf{0}}}}}{\triangle {{{\boldsymbol{MR}}}}}_{{{{\boldsymbol{t}}}}}{{{{\boldsymbol{P}}}}}_{{{{\boldsymbol{t}}}}}+{{{{\boldsymbol{K}}}}}_{{{{\boldsymbol{t}}}}}{{{{\boldsymbol{T}}}}}_{{{{\boldsymbol{t}}}}}{{{{\boldsymbol{L}}}}}_{{{{\boldsymbol{t}}}}}{\triangle {{{\boldsymbol{MR}}}}}_{{{{\bf{0}}}}}{{{{\boldsymbol{P}}}}}_{{{{\bf{0}}}}}}{{{{\bf{2}}}}}\\ 	+\frac{{{{{\boldsymbol{K}}}}}_{{{{\bf{0}}}}}{{{{\boldsymbol{T}}}}}_{{{{\bf{0}}}}}{{{{\boldsymbol{L}}}}}_{{{{\bf{0}}}}}{{{{\boldsymbol{M}}}}}_{{{{\bf{0}}}}}\triangle {{{\boldsymbol{R}}}}{{{{\boldsymbol{P}}}}}_{{{{\boldsymbol{t}}}}}+{{{{\boldsymbol{K}}}}}_{{{{\boldsymbol{t}}}}}{{{{\boldsymbol{T}}}}}_{{{{\boldsymbol{t}}}}}{{{{\boldsymbol{L}}}}}_{{{{\boldsymbol{t}}}}}{{{{\boldsymbol{M}}}}}_{{{{\boldsymbol{t}}}}}\triangle {{{\boldsymbol{R}}}}{{{{\boldsymbol{P}}}}}_{{{{\bf{0}}}}}}{{{{\bf{2}}}}}\\ 	+\frac{{{{{\boldsymbol{K}}}}}_{{{{\bf{0}}}}}{{{{\boldsymbol{T}}}}}_{{{{\bf{0}}}}}{{{{\boldsymbol{L}}}}}_{{{{\bf{0}}}}}{{{{\boldsymbol{M}}}}}_{{{{\bf{0}}}}}{{{{\boldsymbol{R}}}}}_{{{{\bf{0}}}}}\triangle {{{\boldsymbol{P}}}}+{{{{\boldsymbol{K}}}}}_{{{{\boldsymbol{t}}}}}{{{{\boldsymbol{T}}}}}_{{{{\boldsymbol{t}}}}}{{{{\boldsymbol{L}}}}}_{{{{\boldsymbol{t}}}}}{{{{\boldsymbol{M}}}}}_{{{{\boldsymbol{t}}}}}{{{{\boldsymbol{R}}}}}_{{{{\boldsymbol{t}}}}}\triangle {{{\boldsymbol{P}}}}}{{{{\bf{2}}}}}$$

where ∆ represents the change in a factor, and the subscripts *t* and *0* indicate two specific years. The changes in income-based and final sale-based emissions can be decomposed into six socioeconomic drivers in a similar method.

### Drivers in changes of health impacts

In this study, the contributions of determinates to the health risks and lung cancer deaths were determined. Since lifetime lung cancer deaths were obtained through the GEOS-Chem model and lifetime lung cancer risk assessment, not only the socio-economic drivers but also the meteorological change and country-specific lung cancer death between different years can affect the change of PAH-related environmental health risks. The changes in deaths from year *0* to year *t* (∆*D*_0~*t*_) can be decomposed as:

26 $$\triangle {{{{\boldsymbol{D}}}}}_{{{{\bf{0}}}} \sim {{{\boldsymbol{t}}}}}=\triangle {{{{\boldsymbol{D}}}}}_{{{{\boldsymbol{LD}}}}}+\triangle {{{{\boldsymbol{D}}}}}_{{{{\boldsymbol{MC}}}}}+\mathop{\sum}\limits_{{{{\boldsymbol{\theta }}}}}\left({\triangle {{{\boldsymbol{D}}}}}_{{{{{\boldsymbol{Q}}}}}_{{{{\boldsymbol{\theta }}}}}}\right)$$

27 $${\triangle D}_{{Q}_{\theta }}=\frac{1}{2}\left[\left({D}_{{0,Q}_{0}+\triangle {Q}_{\theta }}-{D}_{{0,Q}_{0}}\right)+\left({D}_{t,{Q}_{t}}-{D}_{{t,Q}_{t}-\triangle {Q}_{\theta }}\right)\right]$$

where ∆*D*_*Q*θ_ is the change in deaths caused by the socioeconomic driver of *θ* from year *0* to year *t*; ${D}_{{0,Q}_{0}}$ is the lifetime lung cancer deaths determined by original emissions (*Q*_0_) simulated in the previous year of *0*; ${D}_{{0,{Q}}_{0}+\triangle {Q}_{\theta }}$ refers to the deaths determined with the combination of original emissions (*Q*_0_) and the emissions changed by the socioeconomic driver of *θ* ($\triangle {Q}_{\theta }$) simulated in the year *0*; ${D}_{{t,{Q}}_{t}-\triangle {Q}_{\theta }}$ is the deaths determined with the subtraction of the emissions (${Q}_{t}$) and the emissions changed by the socioeconomic driver of *θ* (∆*Q*_*θ*_) simulated in the year of *t*. To increase the accuracy of the results, forward and backward assumptions were employed through controlling variates. The forward assumption was that only the driver *i* affected the emissions, and the backward assumption was that all the drivers affected the emissions except the driver *θ*. In other words, for each assumption, two simulations were conducted in the same situation (year of *0* or *t*) with different emission maps, and the difference in maps was the addition or removal of emissions caused by the change in a certain socioeconomic driver (determined by SDA). Through controlling variates from two sides, the average of the results represents the influences of the changes in socioeconomic drivers on health impacts.

∆*D*_*MC*_ is the change in deaths due to meteorological changes, which can be determined by taking the average of the results of importing the same emissions into different years (different meteorological data) in the GEOS-Chem model; ∆*D*_*LD*_ is the changes in lifetime lung cancer deaths caused by country-specific lung cancer deaths in different years, which can be obtained by taking the average of the results of importing the same concentrations into different years in the lifetime lung cancer risk assessment. The contributions of meteorological changes and lung cancer deaths can be calculated as:

28 $$\triangle {{{{\boldsymbol{D}}}}}_{{{{\boldsymbol{MC}}}}}=\frac{{{{\bf{1}}}}}{{{{\bf{2}}}}}\left[\left({{{{\boldsymbol{D}}}}}_{{{{\boldsymbol{t}}}},{{{{\boldsymbol{Q}}}}}_{{{{\bf{0}}}}}}-{{{{\boldsymbol{D}}}}}_{{{{\bf{0}}}},{{{{\boldsymbol{Q}}}}}_{{{{\bf{0}}}}}}\right)+\left({{{{\boldsymbol{D}}}}}_{{{{\boldsymbol{t}}}},{{{{\boldsymbol{Q}}}}}_{{{{\boldsymbol{t}}}}}}-{{{{\boldsymbol{D}}}}}_{{{{\bf{0}}}},{{{{\boldsymbol{Q}}}}}_{{{{\boldsymbol{t}}}}}}\right)\right]$$

29 $$\triangle {D}_{{LD}}=\frac{1}{2}\left[\left({D}_{{{LD}}_{t},{{PAF}}_{0}}-{D}_{{{LD}}_{0},{{PAF}}_{0}}\right)+\left({D}_{{{LD}}_{t},{{PAF}}_{t}}-{D}_{{{LD}}_{0},{{PAF}}_{t}}\right)\right]$$

where ${D}_{t,{Q}_{0}}$ is the lung cancer deaths when importing the emission map of previous year *0* to the GEOS-Chem model with the year of *t*; ${D}_{{{LD}}_{t},{{PAF}}_{0}}$ is the deaths when multiplying PAF results of the previous year *0* and the country-specific lung cancer deaths at the year of *t*. Other factors in the equations have similar expressions.

## Supplementary information

Supplementary Information

Supplementary Data 1

Supplementary Data 2

Supplementary Data 3

Supplementary Data 4

Supplementary Data 5

Supplementary Data 6

Supplementary Data 7

Description of Additional Supplementary Files

## Data Availability

The sources of data are provided in Supplementary Data 1. Supporting information (including PAH emission inventory, input-output tables, net flow patterns, and the contributions of different drivers for emissions and health impacts from the perspectives of primary input, final sale, and final consumption) is available at https://zenodo.org/record/6508204.

## Code availability

The GEOS-Chem model (version 13.0.2) source code used in this study is available at: https://zenodo.org/record/4681204.
