# Supplementary material for: Global trade drives transboundary transfer of the health impacts of polycyclic aromatic hydrocarbon emissions
Source: Commun Earth Environ. 2022 Aug 1;3(1):170. doi: 10.1038/s43247-022-00500-y (PMC9340739; doi:10.1038/s43247-022-00500-y)
Supplement: Supplementary file 1 — Supplementary Information [file 43247_2022_500_MOESM1_ESM.pdf]

Supplementary information to

Global trade drives transboundary transfer of the health impacts of polycyclic aromatic hydrocarbon emissions

Ruifei Li[1], Jin Zhang[2, 3, *], Peter Krebs[1]

[1] Institute of Urban and Industrial Water Management, Technische Universität Dresden, 01069 Dresden, Germany

[2] State Key Laboratory of Hydrology-Water Resources and Hydraulic Engineering, Hohai University, 210098 Nanjing, China

[3] Yangtze Institute for Conservation and Development, Hohai University, 210098 Nanjing, China

*Corresponding author:

jin.zhang@hhu.edu.cn

This supplementary information includes:

Supplementary Notes 1-7

Supplementary Note 1: Emission factors with technology splits
Supplementary Note 2: Comparison with other PAH inventories
Supplementary Note 3: Different accountings
Supplementary Note 4: Regional changes and driving factors
Supplementary Note 5: PAH emission linkages analysis
Supplementary Note 6: Key sectors of PAH emissions
Supplementary Note 7: Uncertainty analysis
Supplementary Note 8: Limitations and future research

Supplementary Figures 1-13

Supplementary Figure 1: The framework of different accountings
Supplementary Figure 2: The integrated framework in this study
Supplementary Figure 3: The emissions and deaths occurred in other regions caused by each region's primary inputs, final sales, and final consumption
Supplementary Figure 4: The net flow patterns in 2012
Supplementary Figure 5: The changes in emissions and deaths occurred in each region from 2012 to 2015
Supplementary Figure 6-7: The contributions of global-scale socioeconomic drivers to the emissions and deaths that occurred in each region
Supplementary Figure 8: The sectoral distributions for production-, income-, final sale-, and final consumption-based emissions
Supplementary Figure 9: The uncertainty ranges of production-based, income-based, final sale-base, and consumption-based emissions and lifetime lung cancer deaths of each region in 2015
Supplementary Figure 10: The classification of 13 regions
Supplementary Figure 11: The simulated B*a*P concentrations
Supplementary Figure 12: The locations of the observations
Supplementary Figure 13: Comparison between simulated values and observations

Supplementary Tables 1-6

Supplementary Table 1: Parameters for estimating emission factors
Supplementary Table 2: Parameters of technology split method
Supplementary Table 3: Conceptual framework of the EE-MRIO model in this study
Supplementary Table 4: The net flow patterns of the indicators in 2012 and 2015
Supplementary Table 5: The classifications of sectors
Supplementary Table 6: The summary of emissions and lifetime lung cancer deaths that occurred in each region from 2012 to 2015

Supplementary References

Supplementary Note 1: Emission factors with technology splits

Sixteen priority PAHs were included in the inventory, which are naphthalene (NAP), acenaphthylene (ACY), acenaphthene (ACE), fluorene (FLU), phenanthrene (PHE), anthracene (ANT), fluoranthene (FLUH), pyrene (PYR), benzo[*a*]anthracene (B*a*A), chrysene (CHR), benzo[*b*]fluoranthene (B*b*F), benzo[*k*]fluoranthene (B*k*F), benzo[*a*]pyrene (B*a*P), indeno[*1,2,3-cd*]pyrene (IDP), dibenzo[*a,h*]anthracene (DBA), and benzo[*g,h,i*]perylene (B*g*P). The PAH emission factors varied because of the development of science and technology over time. Each source of PAHs can be divided into several technology divisions by using the technology split method. This method assumes that emission factors for each technology division keep constant, and fractions of various technology divisions changed over time. The equation of the technology split method is:

$$Y(t) = (Y_0 - Y_f)e^{(-(t-t_0)^2/2s^2)} + Y_f \quad \text{(S1)}$$

where Y_0 and Y_f represent initial and final fractions of a certain technology division, respectively; t_0 indicates the start time of the technology transition, and *s* is a rate.

The emission factors for transportation sectors also varied over time. Based on GDP per capita (1000 USD), the monovariate regression models were employed to predict the 16 PAHs emission factors (mg/t) from different vehicle emissions. The equation can be expressed as:

$$\log(EF_{icy}) = k_i * GDP_{ccy} + C_i \quad \text{(S2)}$$

where EF_{icy} (mg/t) indicates the emission factor of compound *i* for *y* model year

vehicles produced in a certain country *c*; k_i indicates the model slope of compound *i* ; C_i is the interception of compound *i*; GDP_{ccy} indicates the GDP per capita of a given country *c* in year *y* in 1000 USD. The details about emission factors are presented in Supplementary Tables 2-4 [1].

Supplementary Note 2: Comparison with other PAH inventories

This study estimated the 16PAHs emissions emitted in the world from 2012 to 2015. The emission inventory in this study includes sources of fuel consumption (including coal, natural gas, petroleum, and biomass), and industrial processes (including primary Al production, pig iron production, crude steel production, coke production, petroleum catalytic cracking, and agricultural residues burning). The natural sources were calculated, but not involved in the EE-MRIO model. The PAH emission inventory in 2015 was estimated as 357 Gg (anthropogenic sources: 304 Gg). Several previous studies estimated the PAH emissions from different sources in different years. Zhang and Tao [2] investigated the global PAH emission inventory (530 Gg) for 2004 and reported the anthropogenic emission as 440 Gg in which constant emission factors were adopted. Considering the different emission factors, sources and years, this result was relatively consistent with this study. Shen, et al. [1] updated the PAH emission factors using the technology split method and the monovarietal regression models and estimated the global PAH emissions as 507 Gg in 2007. They also projected the PAH emission inventory from 2008 to 2030, and their estimated inventory for 2015 was around 350 Gg (uncertainty range: 250~620 Gg). Since the method for calculating the emission factors, emission sources, and fuel consumption data were the same, our

results (357 Gg) were almost equal to their estimated inventory.

From the perspective of regional emissions, the emission inventories for 13 regions in 2015 in this study were: CA: 3.0 Gg, CN: 77.8 Gg, EE: 9.3 Gg, IN: 47.9 Gg, LA: 28.3 Gg, MN: 19.9 Gg, RA: 52.6 Gg, RE: 2.9 Gg, RW: 3.5 Gg, RU: 6.4 Gg, SS: 88.1 Gg, US: 6.1 Gg, WE: 11.5 Gg. The regional emission inventories in 2007 in the literature were: CN: 106 Gg, IN: 67 Gg, US: 8.5 Gg, and RU: 8.6 Gg [1]. Considering the decreasing emissions from 2007 to 2015, the regional emission inventories were relatively consistent with the previous study [1]. The uncertainty of emission inventory is the uncertainty of production-based emissions discussed in **Part S7** and presented in **Fig. S7**.

Supplementary Note 3: Production-, income-, final sale-, and final consumption-based accountings

This study investigated the influence of international trade on PAH emissions and health impacts from the whole stages of the supply chain (primary input, production, final sale, and final consumption).

Supplementary Figure 1 shows the framework of production-based, income-based, final sale-based, and final consumption-based accounting. There are four kinds of regions in a supply chain (Supplementary Figure 1): region A is the primary supplier whose investment supports the downstream production process, region B is the producer of products with direct emissions, and region C is the final seller of products (or say the final producer), and region D is the final consumer. Regions A, C, and D have no direct emissions, but they all benefit from production processes. Primary suppliers (e.g., supplies of capital and labor forces) receive a payment from the inputs

that they supply. Final sellers (producers) profit from the sales of final products. Final consumers benefit from the enjoyment of products and services. Using the income-based, final sale-based, and final consumption-based accounting methods, the PAH emissions directly emitted by producers can be allocated to different roles in the supply chain as primary suppliers, final sellers (final producers), and final consumers. The total amounts of emissions remain the same but the allocations of emissions among different worldwide regions are determined by different methods.

Moreover, income-based, production-based, final sale-based and consumption-based accounting frameworks measure the relative importance and contributions of regions as the primary supplier, direct emitters, final sellers (final producers), and final consumers, respectively, to total emission flows. They describe a region's role within supply chains and identify critical regions for the implementation of different types of measures (as shown in Supplementary Figure 1).

These accounting methods were based on the multiregional input-output model. Supplementary Table 1 shows the conceptual framework of the environmental extended multiregional input-output model (EE-MRIO) in this study. Notations in Supplementary Table 5 are explained as follows:

(1) The superscripts m, n indicate the region at row and column, and the superscripts i, j represent the sector at row and column.

(2) Z_{ij}^{mn} indicates the intermediate delivery of products from sector i in region m to sector j in region n.

(3) f_i^{mn} indicates the final demand of region n for the products from sector i in region

m.

(4) v_j^n indicates the primary inputs (added values) of sector *j* in region *n*.

(5) X_i^m indicates the total output of sector *i* in region *m*, and X_j^n represents the total input of sector *j* in region *n*.

For the income-based accounting, the income-based emission of sector *j* in region *n* is calculated by $Q_j^n = V_j^n (I - B)^{-1} U$, and $V_j^n = [0 \quad \cdots \quad v_j^n \quad \cdots \quad 0]$. For the income-based results matrix Q_I calculated by $Q_I = V(I - B)^{-1} U$ and $\mathrm{v} = [v_1^1 \quad \cdots \quad v_j^1 \quad \cdots \quad v_j^n]$ (Equation 14 and 12 in Method 4.3), the sum of the elements in the *m* row is income-based emissions of sector *i* in region *m*, and the sum of those in the *n* column is production-based emissions of sector *j* in region *n*.

For the final sale-based accounting, the final sale-based emission of region *m* is calculated by $Q^m = U(I - A)^{-1} Y^m$ and $Y^m = \begin{bmatrix} 0 \\ \vdots \\ \sum_n f_i^{mn} \\ \vdots \\ 0 \end{bmatrix}$. $\sum_n f_i^{mn}$ refers to the sum of total products sold from region *m* to all destination regions. For the income-based results matrix Q_S calculated by $Q_S = U(I - A)^{-1} Y$ and $\mathrm{y} = \begin{bmatrix} \sum_n f_1^{1n} \\ \vdots \\ \sum_n f_i^{1n} \\ \vdots \\ \sum_n f_i^{mn} \end{bmatrix}$ (Equation 15 and 11 in Method 4.3), the sum of the elements in the *m* row is the production-based emissions of sector *i* in region *m*, and the sum of those in the *n* column is the final sale-based emissions of sector *j* in region *n*.

For the final consumption-based accounting, the final consumption-based

emission in region *n* is calculated by $Q^n = U(I-A)^{-1}F^n$ and $F^n = \begin{bmatrix} f_1^{1n} \\ \vdots \\ f_i^{1n} \\ \vdots \\ f_i^{mn} \end{bmatrix}$. F^n refers to the total final demand of region *n* for the products from all sectors in all regions. For the final consumption-based results matrix Q_C calculated by $Q_C = U(I-A)^{-1}F$ and $F = \begin{bmatrix} f_1^{11} & f_1^{12} & \cdots & f_1^{1n} \\ f_2^{11} & f_2^{12} & \cdots & f_2^{1n} \\ \vdots & \vdots & & \vdots \\ f_i^{11} & f_i^{12} & \ddots & f_i^{1n} \\ \vdots & \vdots & & \vdots \\ f_i^{m1} & f_i^{mn} & \cdots & f_i^{mn} \end{bmatrix}$ (Equation 16 and 10 in Method 4.3), the sum of the elements in the *m* row is the production-based emissions of sector *i* in region *m*, and the sum of those in the *n* column is the final consumption-based emissions of sector *j* in region *n*.

Supplementary Note 4: Regional changes and driving factors

Supplementary Figure 5 shows the changes in PAH emissions and lifetime lung cancer deaths that occurred in each region from 2012 to 2015. In this period, the emissions in China and India had larger declines, while emissions of Sub-Saharan Africa increased significantly. For lifetime lung cancer deaths, the increase in China was larger than that of other regions. From the temporal perspective, although the health impacts kept increasing during this period, the decrease in emissions of populous regions can mitigate the health impacts. For instance, from 2013 to 2014, the emissions emitted in China declined greatly, contributing to 1% of the decrease in the global PAH emissions. Meanwhile, the increase in health impacts in China was the lowest in this period with a large decrease in emissions. Thus, the mitigation of emissions still can reduce the PAH-related health impacts, especially in populous regions.

The contributions of different factors to the changes in PAH emissions and lifetime lung cancer deaths that occurred in each region from 2012 to 2015 are presented in Supplementary Figures 6 and 7**.** From 2012 to 2015, emissions in most regions were reduced, except in several regions (such as Sub-Saharan Africa (SS) and the Middle East and north Africa (MN)). In terms of the driving factors, the decrease in the emissions of China was mainly caused by the decreasing emission factor attributed to the improvement of environmental protection measures. Meanwhile, the decrease in input/sale/consumption level drove the decline in emissions of India and the rest of Asia. In addition, the increases in emissions of most regions were caused by declines in energy efficiency and input/sale structure. However, the situation in China was different.

Due to the large economic development, the energy efficiency resulted in a decrease in emissions. Since the large export of products, the input/sale structure and consumption level caused the most increase in emissions of China. Thus, it is essential to improve the economic supply chain through global cooperation for mitigating PAH emissions.

In terms of health impacts, meteorological change was the most significant factor driving the increase in health impacts in most regions. The decrease in health impacts of PAH caused by reducing emissions was offset by meteorological change. Furthermore, energy efficiency drove the large increases in deaths in India, the rest of Asia, and Sub-Saharan Africa. With the increase in lung cancer rate in the world, the increase in PAH-related deaths was more serious in the world, especially in populous regions. Thus, the mitigation of PAH-related health impacts requires both the improvement of energy efficiency in developing regions and the optimization of the global economic supply chain.

Supplementary Note 5: PAH emission linkages analysis

In this study, emission linkage analysis was used to identify economic sectors' total polluting characteristics based on intersectoral linkages of PAH emissions [3]. Emission linkage analysis can determine the key PAH emission sectors with the greater potential to reduce PAH emissions through small changes in economic activity [4,5]. The generalized backward and forward linkages (BL, FL) can be obtained using the following equations:

$$\boldsymbol{BL_j} = \frac{\mu_j}{\sum_{j=1}^{n} \mu_j / n}, \qquad \text{(S3)}$$

$$FL_j = \frac{\vec{\mu}_i}{\sum_{i=1}^{n} \vec{\mu}_i / n}. \quad \text{(S4)}$$

In the analysis, $l = E(I - A)^{-1}$ indicates the total embodied emission intensity, whose element l_{ij} can be applied to calculate $\mu_j = \sum_{i=1}^{n} l_{ij}$, representing the backward emission multiplier of sector i, and $g = (I - b)^{-1}E$ indicates the total enabled emission intensity, whose element g_{ij} can be used to calculate $\vec{\mu}_i = \sum_{j=1}^{n} g_{ij}$, representing the forward emission multiplier of sector i [6]. If $BL_j > 1$, a monetary unit increase in the final demand of sector j will cause an above-average increase in PAH emissions. If $FL_i > 1$, a monetary unit increase in the primary input of sector i will cause an above-average increase in PAH emissions. Both BL_j and FL_i are larger than one, indicating that they can be greatly affected by the final demands and primary inputs related to PAH emissions, and thereby there are determined as the key emission sectors.

Supplementary Note 6: Key sectors of PAH emissions

By applying the emission linkage analysis based on PAH emission multipliers, the key emission sectors for 2015 were determined. There are a lot of identified key sectors determined because of a lot of involved regions. The common key sectors among the regions were *Petroleum, Chemical, and Non-Metallic Mineral Products*, *Metal Products*, *Transport, Commercial services,* and *Private household*. For the sector of *Petroleum, Chemical, and Non-Metallic Mineral Products*, this sector contains coke production and many types of petroleum fuel consumption, which emitted a large amount of PAH emissions. For the sector of *Metal Products*, this sector involves iron, steel, and aluminum production, whose process can release a lot of PAHs. And the products of these two sectors can be utilized as input for other sectors, playing important

role in international trade. For the sector of *Transport*, this sector includes most of the emissions from diesel and gasoline combustions of vehicles. Transportation is required for most of the products involved in the international trade, and indirectly causes the release of PAH emissions in other regions. The sector of *Private household* consumed a lot of biofuels for burning, which is an important source of PAHs. The role of this sector is mainly the final consumer, which requires the products supplied from other sectors and regions. The products, functions, or final demands of these sectors are strongly linked with international trade, which enables the emissions of the sectors to transfer or distribute on a global scale. In addition, the fuel combustion in private households can also emit a lot of PAHs. These high-priority sectors are important for the mitigation of PAH emissions as a decrease in the final demands or primary inputs of these sectors can result in a greater reduction in PAH emissions than such decreases in other sectors. Therefore, it is important to understand the consumption and supply patterns of these sectors to achieve comprehensive insights into PAH pollution control.

This study mainly focused on the influences of international trade on global PAH emissions, so the global MRIO tables were applied. The sectors in these tables were less than the national input-output tables. Therefore, it is recommended to apply the national input-output tables and emission linkage analysis to determine the detailed key sectors for a certain country. For example, the contributions of different sectors to mercury emissions in China have been analyzed through Chinese input-output tables in 2010 [7]. Zhang, et al. [8] applied emission linkage analysis and Chinese input-output tables to determine the key sectors for mercury emissions in China. Furthermore, seven

Chinese key sectors for PAH emissions from 2002 to 2017 (*coking industry; steel, iron, and iron alloy products; non-ferrous metal smelting industry; road transportation; air transport industry; warehousing;* and *water management*.) have been determined by a previous study [9]. Compared with the Chinese key PAH sectors, the common global key sectors determined in this study were involved in their results. Therefore, the results of the key sectors are reliable.

Supplementary Note 7: Uncertainty analysis

Our results are subject to several uncertainties, and we estimated the uncertainty ranges in different steps of our analysis, including the compilation of the global PAH emission inventory, the EE-MRIO model, and the simulation of the GEOS-Chem chemical transport model, the lifetime lung cancer risk assessment. The overall uncertainties of production-based, income-based, final sale-based, and consumption-based PAH emissions, environmental health risks, and lifetime lung cancer deaths are derived from aggregations of the uncertainties above using Monte Carlo simulation at 10,000 samplings. The P5 and P95 values of the statistical distribution curves were set as the lower and upper limits of the uncertainty range following the previous studies (Supplementary Figure 9) [7,10]. The detailed uncertainty analysis for different step are as follows:

(1) The compilation of global PAH emission inventory is subject to uncertainty due to incomplete knowledge of fuel consumption, emission factors, and activity rates. The uncertainty of source-specific PAH emission factors was obtained from previous literature [1]. The uncertainties of fuel consumption and activity rates are not provided in

the statistics of the International Energy Agency (IEA). Following the previous study [1], the various intervals of historical fuel consumption and activity rates were set to be 20% of the means for biomass burning and open fires, 5% for energy production, 30% for waste burning, 15% for ship and aviation and industrial sectors, and 10% for all other sources. Thus, the uncertainty of region-specific production-based PAH emissions can be obtained based on the uncertainties of emission factors, fuel consumption, and activity rates.

(2) The estimation of income-based, final sale-based and consumption-based PAH emissions of worldwide regions share the most uncertainties with production-based PAH emissions and includes an additional uncertainty from the EE-MRIO model associated with the inaccuracies of sectoral mapping, economic statistics, and data harmonization [11-13]. A previous study investigated a comparable variability between production-based and consumption-based CO_2 emissions across studies using different MRIO models and reported that MRIO-related uncertainty was relatively smaller than the uncertainty of production-based emissions [14]. According to previous study [14], the uncertainty for the past estimates of global CO_2 emissions embodied in trade is suggested to vary up to 13 %. In this study, a 13 % uncertainty to production-based PAH emissions was added to represent the uncertainty of income-based, final sale-based, and consumption-based PAH emissions derived from the EE-MRIO model, consistent with previous studies such as those by Lin, et al. [15], Chen, et al. [7] and Zhang, et al. [10].

(3) Atmospheric PAH concentrations simulated by the GEOS-Chem chemical

transport model can be influenced by the uncertainties of emission input maps and the model representation of physical and chemical processes, including vertical transport, gas-particle partition, deposition, and scavenging. Due to the large computational intensity of the GEOS-Chem chemical transport model, it is infeasible to conduct uncertainty analysis through sensitivity analysis or Monte Carlo simulations, requiring many model runs. According to the method applied in the previous studies [7,10], we applied the normalized root-mean-square deviation (NRMSD) between the observed and simulated atmospheric PAH concentrations to represent the uncertainty of the GEOS-Chem chemical transport model. The ground observations are from European Monitoring and Evaluation Programme (EMEP) (www.projects.nilu.no/ccc/emepdata, assessed Oct. 2020), and the National Air Pollution Surveillance Network of Canada (NAPS) (www.data.ec.gc.ca, assessed Mar. 2021), and measurements in the world from literature (Supplementary Table 12).

(4) The lifetime lung cancer deaths were obtained by lifetime lung cancer risk assessment. According to the equations for calculating risks, the uncertainties of environmental health risks are attributed to the uncertainties of PAH concentrations and URR values. For each scenario, the uncertainties of PAH concentrations are aggregated by the uncertainties of production-based emissions, EE-MRIO model, and GEOS-Chem chemical transport model. The uncertainty range of URR values varied between regions and can be obtained from the literature [16]. Based on the equations for calculating the lifetime lung cancer deaths, the uncertainties of lifetime lung cancer deaths can be affected by the uncertainty of PAF, country-specific lung cancer rates (1 death per

100,000 people), and population. The uncertainty range of country-specific lung cancer rates can be obtained from the Global Burden of Disease (GBD) database (www.healthdata.org, assessed Mar.2021). Since the uncertainty of population is not provided in the statistics, we assume a 10% uncertainty for the world population according to the previous studies [7,10]. After the Monte Carlo simulations with 10,000 trails of PAH concentrations, URR values, country-specific lung cancer rates, and population, the uncertainties of environmental health risks and lifetime lung cancer deaths were determined.

Since the SDA is used to quantify the contributions of different drivers to the changes in emissions and health impacts, the percentages of drivers are proportional to the changes in emissions and health impacts. Thus, the percentages of drivers do not have uncertainty ranges. Therefore, following the previous study [17], the uncertainty of the contribution percentages is not discussed in this study.

Supplementary Note 8: Limitations and future research

In addition to the uncertainties discussed above, there are several limitations in this study. For the validation of the model, it can be found that there is a relatively small difference between observed concentrations and the simulated concentrations from the chemical transport model. There are several reasons for the difference. First, the simulated concentrations were the annual average values, while the observed concentrations were taken from some samples for certain days. If more measuring stations can be built to measure the daily or weekly PAH concentrations, the observed concentrations can be more representative of this region's annual average pollution

level. Second, the emissions might be underestimated or overestimated due to the uncertainties of emission factors and activity rates. The simulation can be improved as better statistical data, accurate emission factors, and monitoring data become available in the future.

Furthermore, the long-range transport model can also be improved by considering phase separation and organic aerosol viscosity-dependent shielding. Shrivastava, et al. [18] investigated the global long-range transport and lung cancer risk from PAHs shielded by coatings of organic aerosol. Particle-bound BaP was found to degrade in a few hours by heterogeneous reaction with ozone in laboratory measurements, but BaP persists much longer in the atmospheric field observations. They found that the shielding of BaP from oxidation by coatings of viscous organic aerosol might be the reason for the phenomenon. This organic aerosol viscosity-dependent shielding, which varies with temperature and humidity, can assist a global transport model to obtain better predictions of PAHs. Organic aerosol coatings strongly modulate the atmospheric persistence of PAHs and their cancer risks. Zhou, et al. [19] found that phase separation plays an important role in the ozonolysis of PAHs mixed with secondary organic aerosols and organic oils based on the experiments combined with state-of-the-art kinetic and thermodynamic models. Ozonolysis products of the PAHs phase separate to form viscous surface crusts, which can protect underlying PAHs from ozonolysis to prolong their chemical lifetime. Their results indicated that phase separation and slow diffusion can affect the long-range transport of PAHs in the atmosphere and their fates in indoor environments by prolonging the chemical lifetime of PAHs. Due to the

limitation of the GEOS-Chem model, the simulated concentration of BaP might be underestimated. In future research, phase separation and coatings of viscous organic aerosol can be applied in the global transport model to improve the simulation.

This study mainly focuses on the relationship between the economic supply chain and PAH emissions and health impacts from outdoor exposure. The chemical transport model simulated the ground-level PAH concentration based on different emission inputs. However, several previous studies reported that indoor exposure to PAH pollution was also quite important to people's health, especially for the cooking process [20,21]. In future research, the trends in the indoor PAH-related health impacts can further be investigated. In addition, not only the economic supply chain but also land-use change can affect the emissions and pollution-related health impacts [22,23]. Previous studies investigated the trends and drivers of GHG emissions from land management and land-use change [22]. The relationship between PAH health impacts and land-use changes can be further investigated in the future.

The results of this study show that the Asia regions, especially in China, emitted a large amount of PAH pollution, resulting in a large number of lifetime lung cancer death due to the large population in the regions. Although there are proportions of pollution and deaths attributed to other regions' primary inputs, final sales, and final consumption, detailed mitigation strategies in these regions should be done. A previous study has investigated the consumption-based and income-based sectoral PAH emissions in China from 2002 to 2017 [9]. However, the whole supply chain-based PAH health impacts in China are still unknown. Furthermore, it is vital to gain a deep

understanding of the socioeconomic drivers for the long-term PAH-related health impacts in China. In the future, with the improvement of the chemical transport model, the influences of interregional and international trade on PAH-related health impacts with fine resolution simulation in a certain region, such as China, can be further investigated to optimize the production, primary input, final sale, and consumption structures for mitigating PAH emissions and health impacts.

Supplementary Figure 1: The framework of production-based, income-based, final sale-based, and final consumption-based accounting.

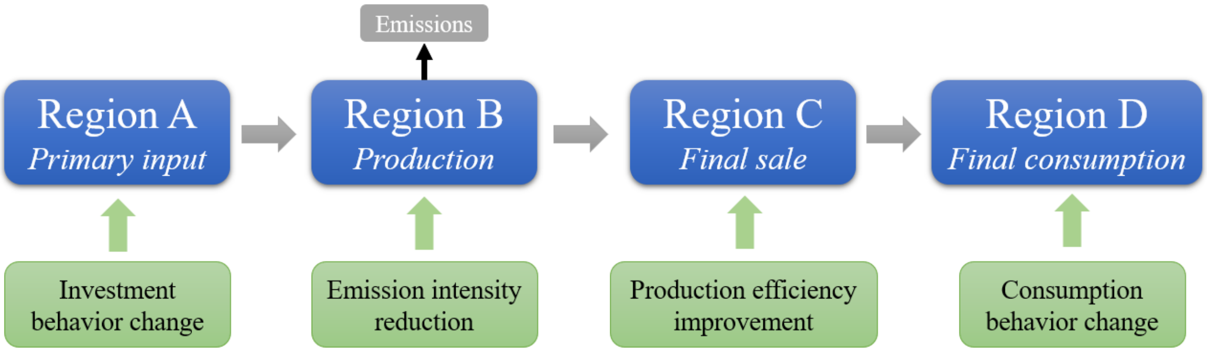

Frameworks	Region A	Region B	Region C	Region D
Income-based	Emissions	0	0	0
Production-based	0	Emissions	0	0
Final sale-based	0	0	Emissions	0
Final consumption-based	0	0	0	Emissions

Supplementary Figure 2. The integrated framework in this study.

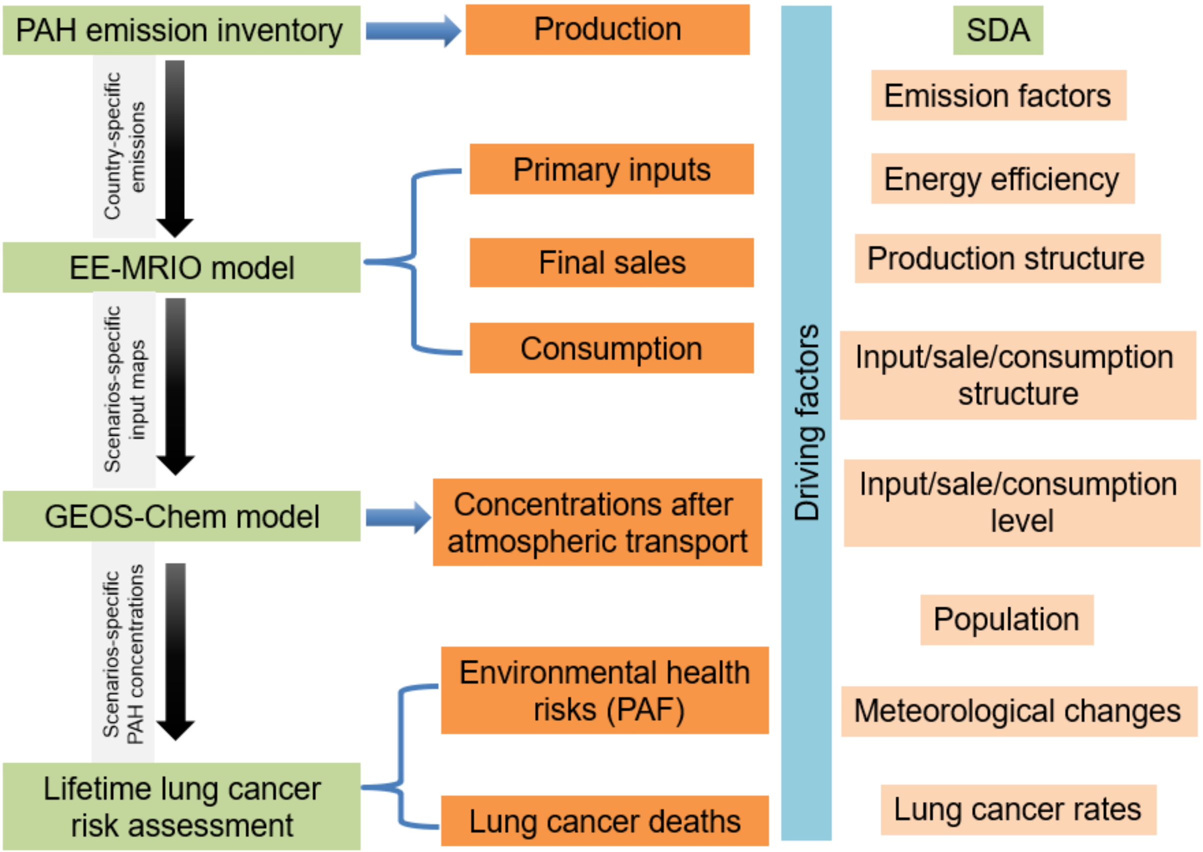

Supplementary Figure 3. The emissions and lifetime lung cancer deaths occurred in other regions caused by each region's primary inputs, final sales, and final consumption.

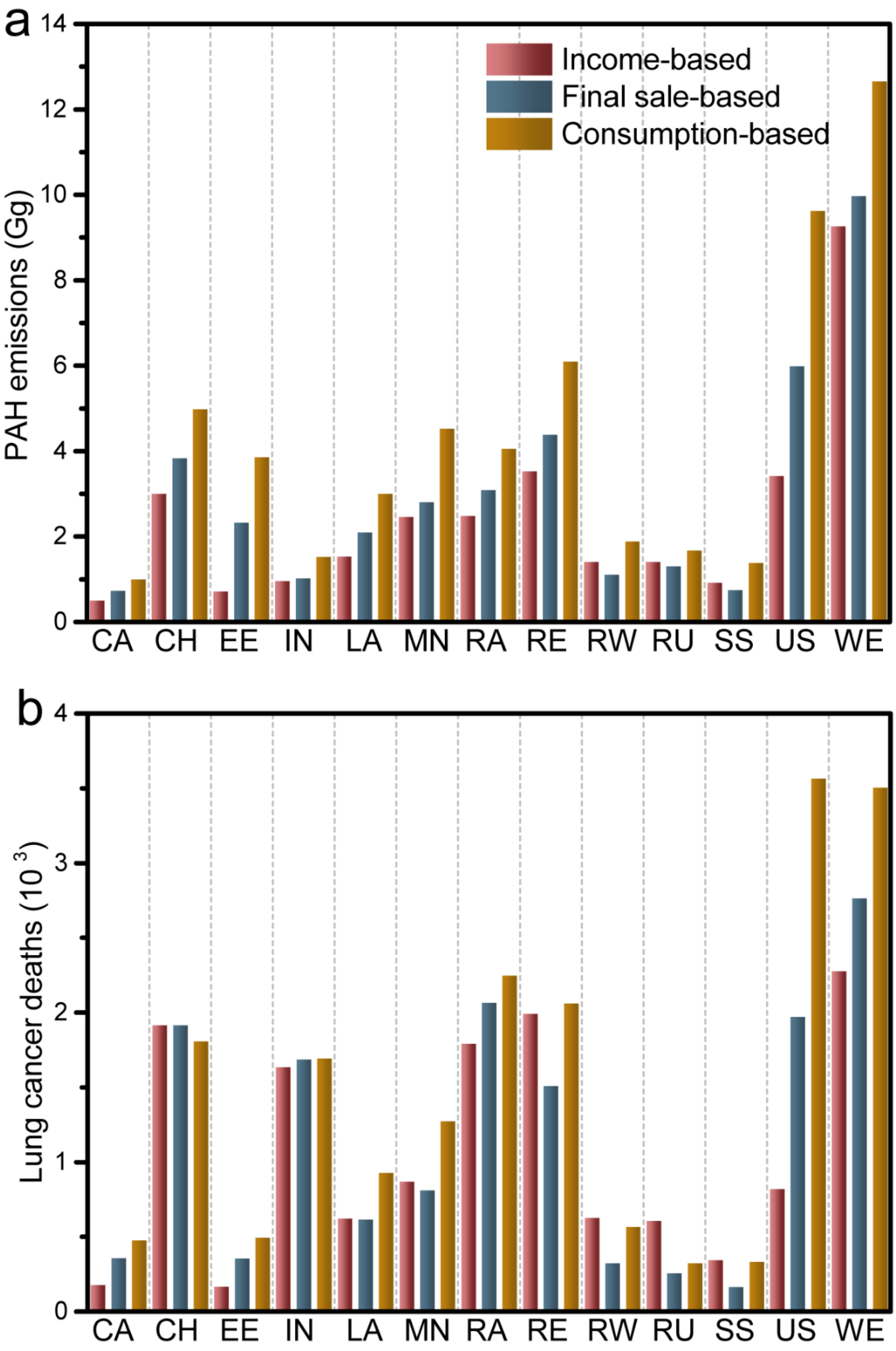

Supplementary Figure 4. The net flow patterns of income-based (**a**, **b**, **c**), final sale-based (**d**, **e**, **f**), and consumption-based (**g**, **h**, **i**) PAH emissions and lifetime lung cancer deaths in 13 regions in 2012.

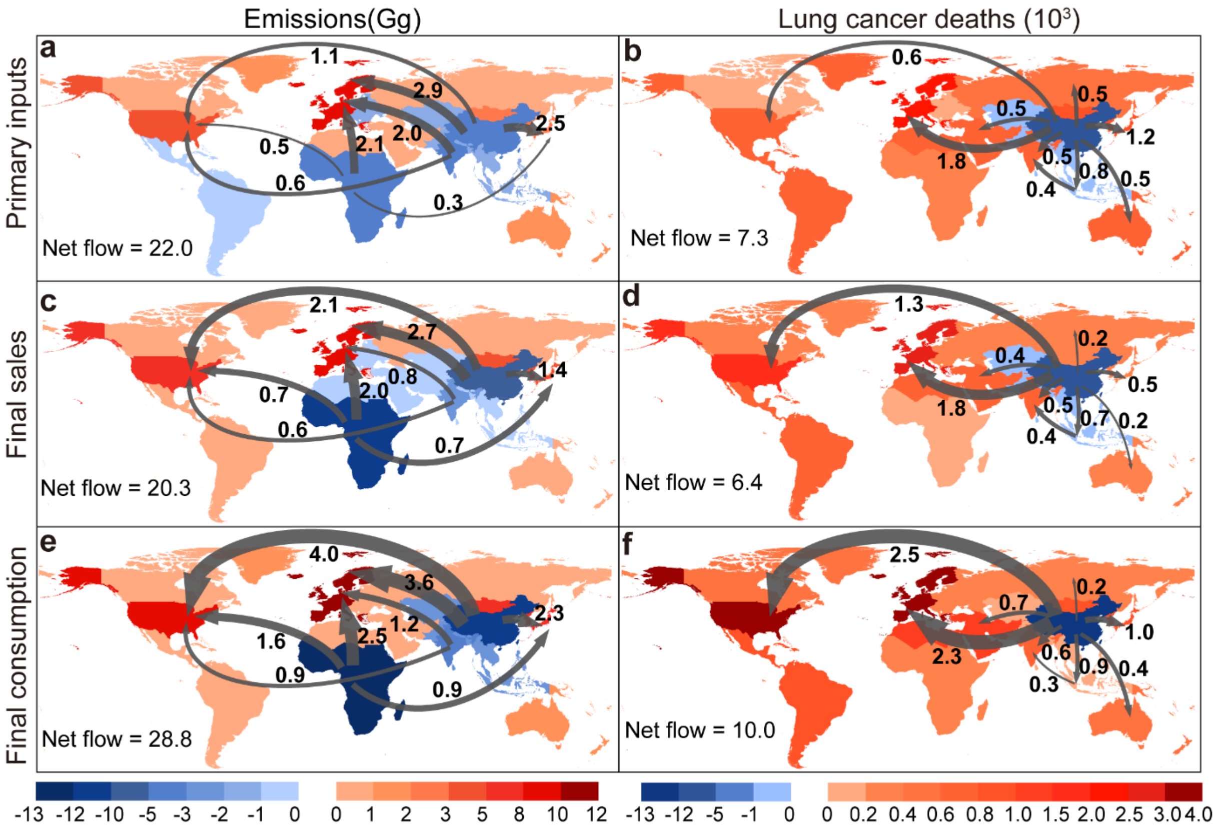

Supplementary Figure 5. The changes in emissions and deaths occurred in each region from 2012 to 2015.

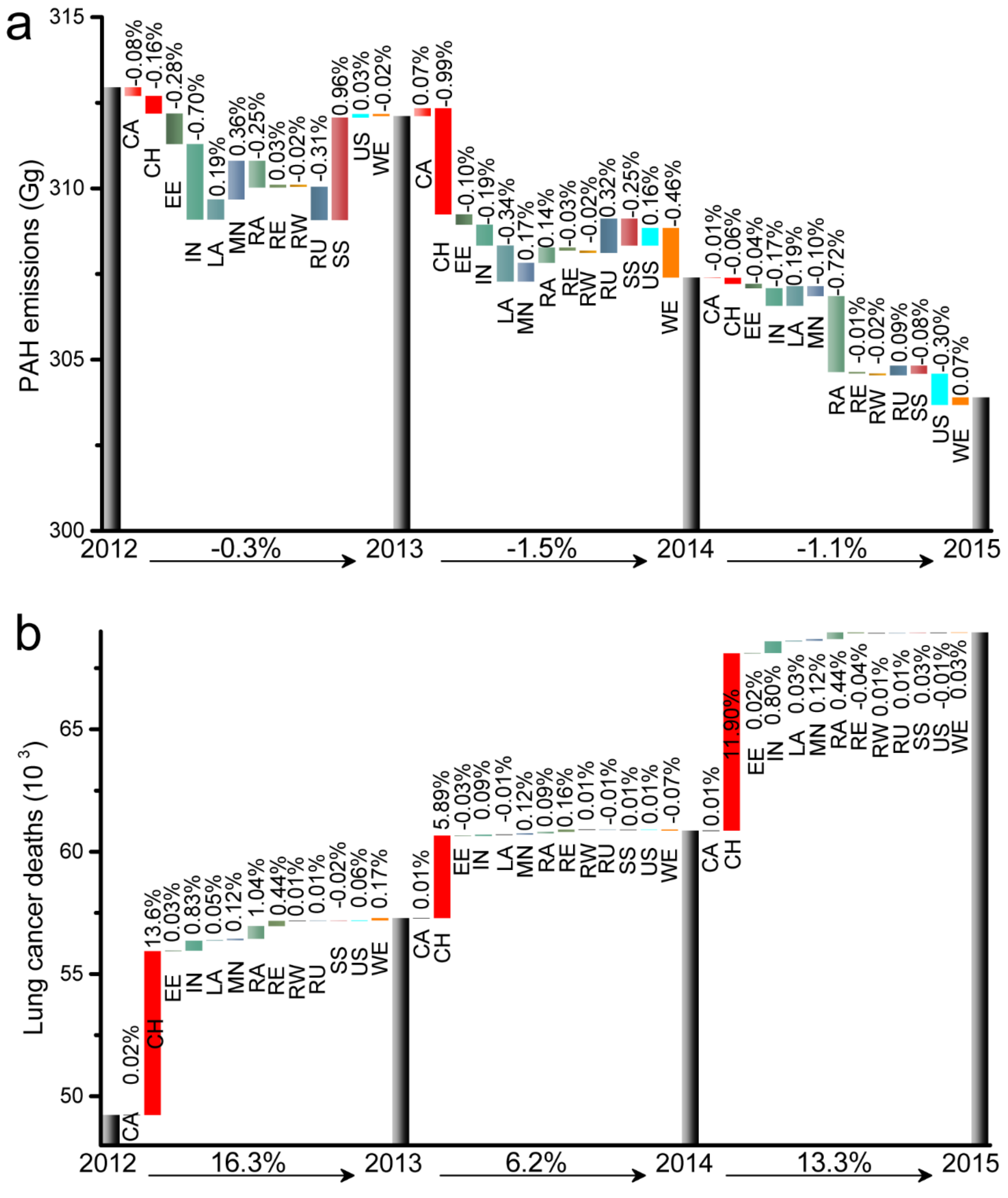

Supplementary Figure 6. The contributions of global-scale socioeconomic drivers to the emissions occurred in each region from the perspective of primary inputs(**a**), final sales(**b**), and final consumption (**c**) from 2012 to 2015.

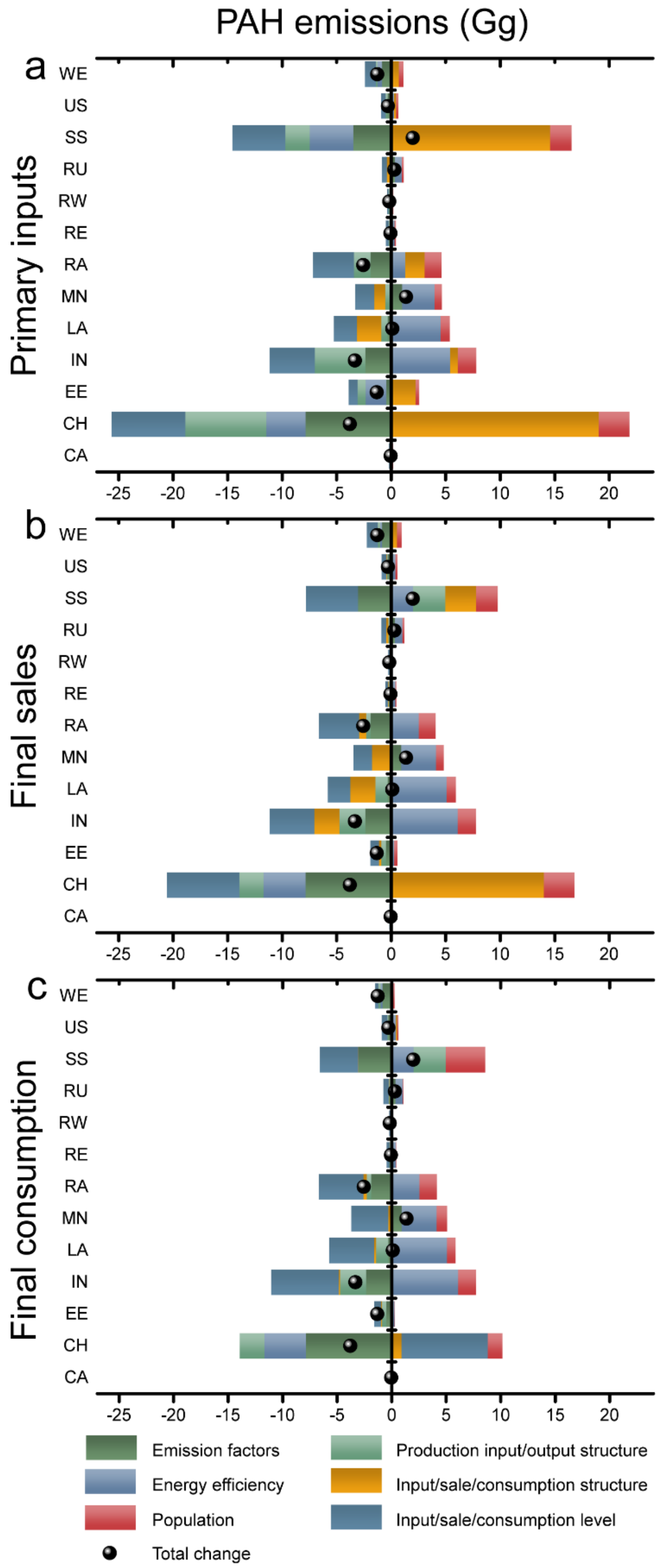

Supplementary Figure 7. The contributions of global-scale socioeconomic drivers to the lifetime lung cancer deaths occurred in each region from the perspective of primary inputs(**a**), final sales(**b**), and final consumption (**c**) from 2012 to 2015. Due to a large number of deaths in China, it is shown in a single figure (**d**).

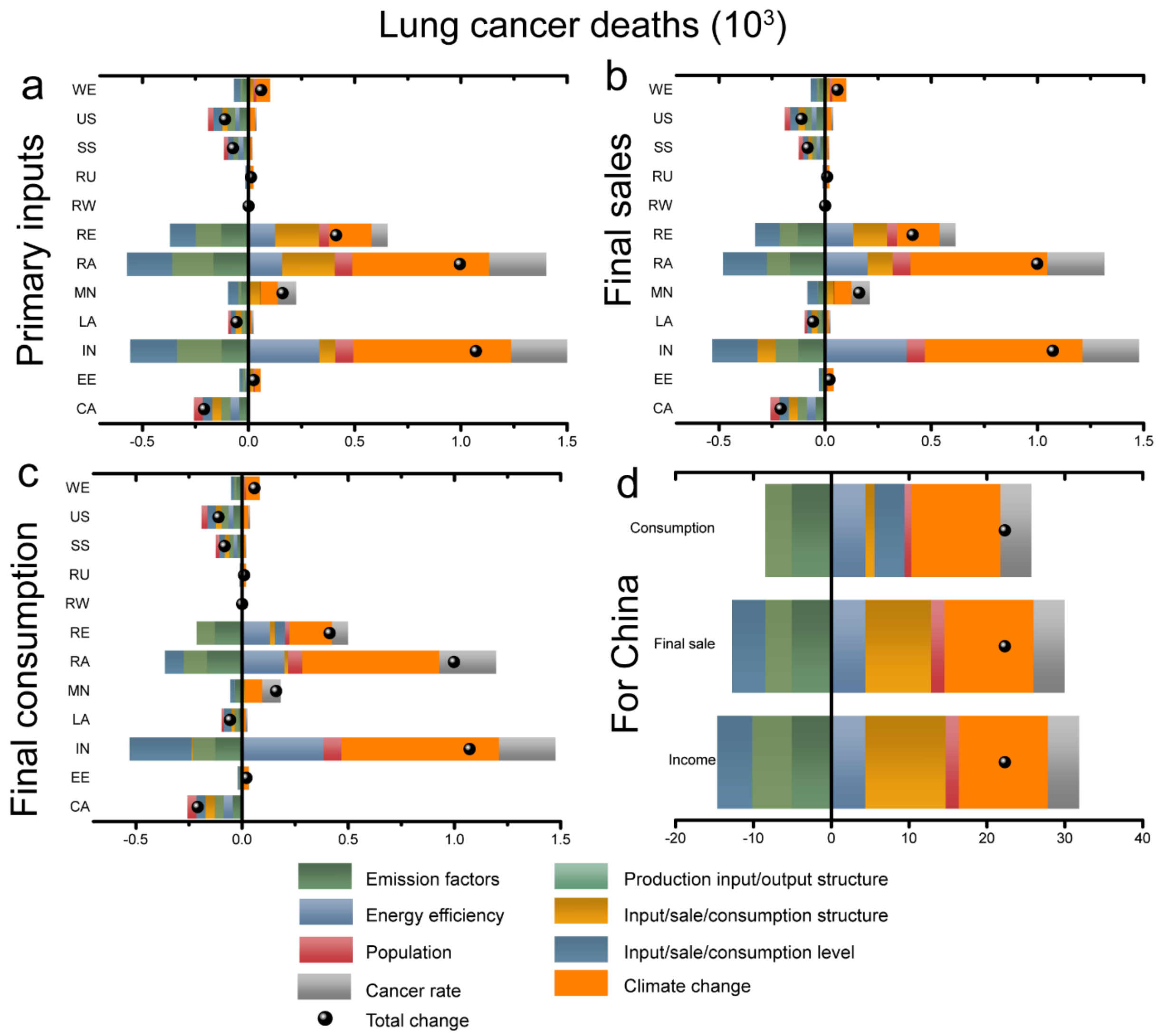

Supplementary Figure 8. The sectoral distributions (%) in each region: (a) production-based emissions which were directly emitted by different sectors in each region; (b) consumption-based emissions which were driven by one region's final demand and emitted by different sectors in all regions, (c) final sale-based emissions which were driven by each sector's final sale, (d) income-based emissions which were driven by each sector's primary input.

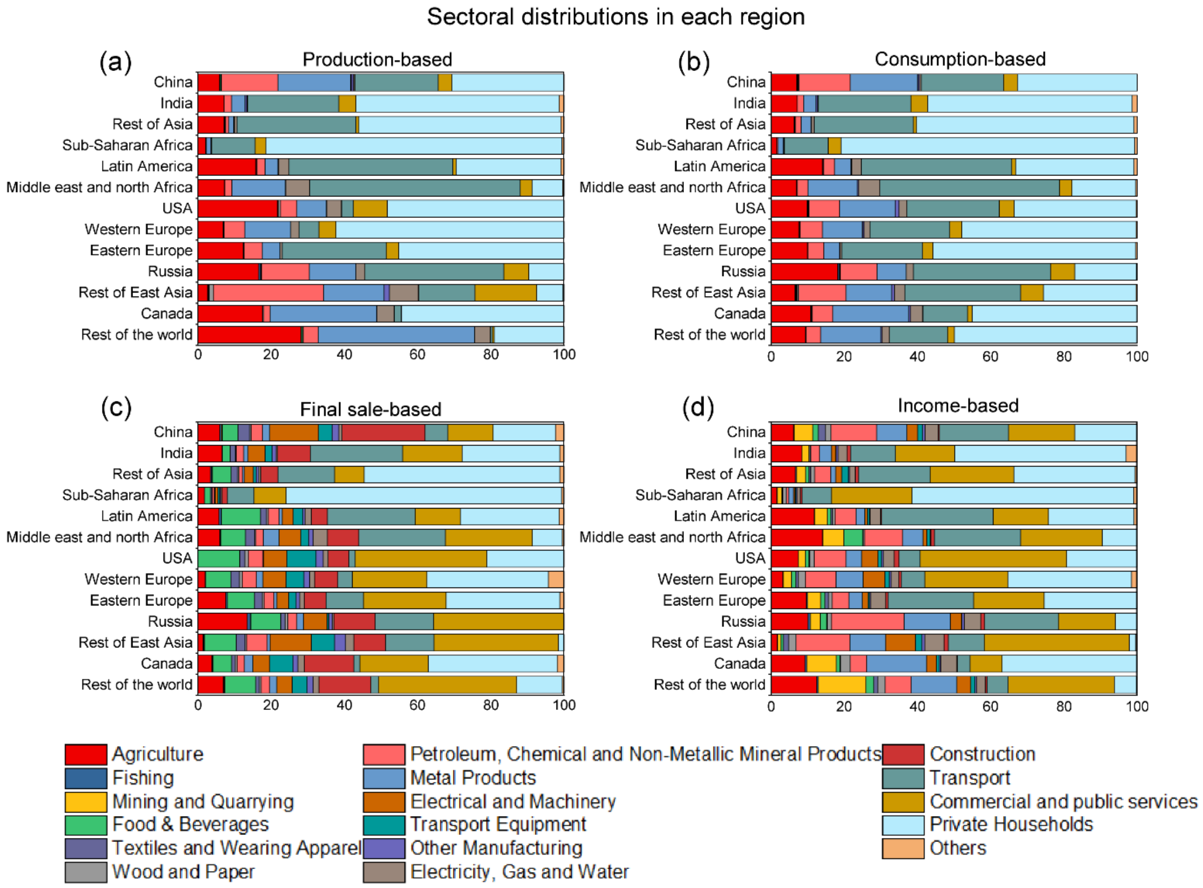

Supplementary Figure 9. The uncertainty ranges (95% confidence interval) of production-based, income-based, final sale-base and consumption-based emissions and lifetime lung cancer deaths of each region in 2015.

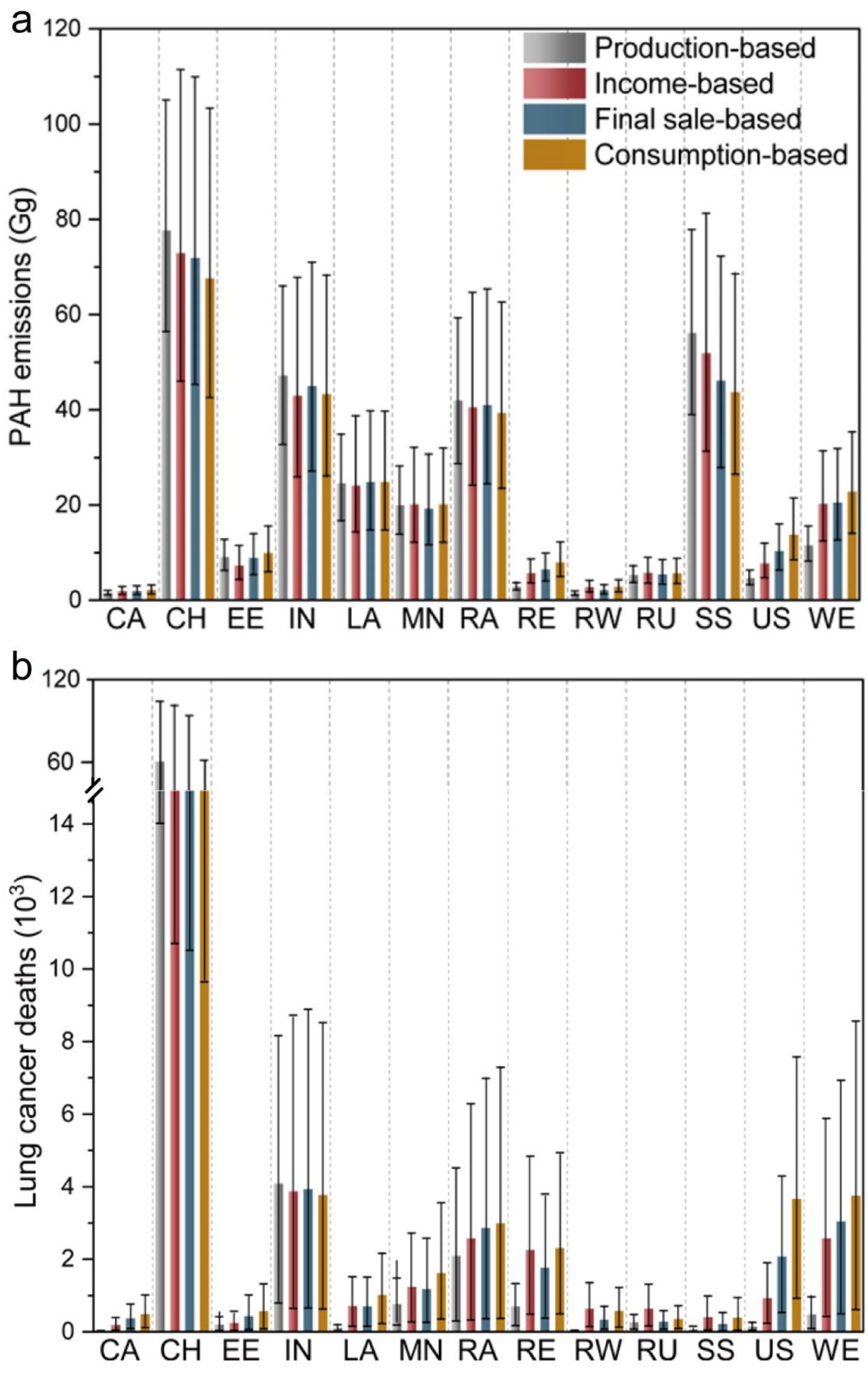

Supplementary Figure 10. The classification of 13 regions.

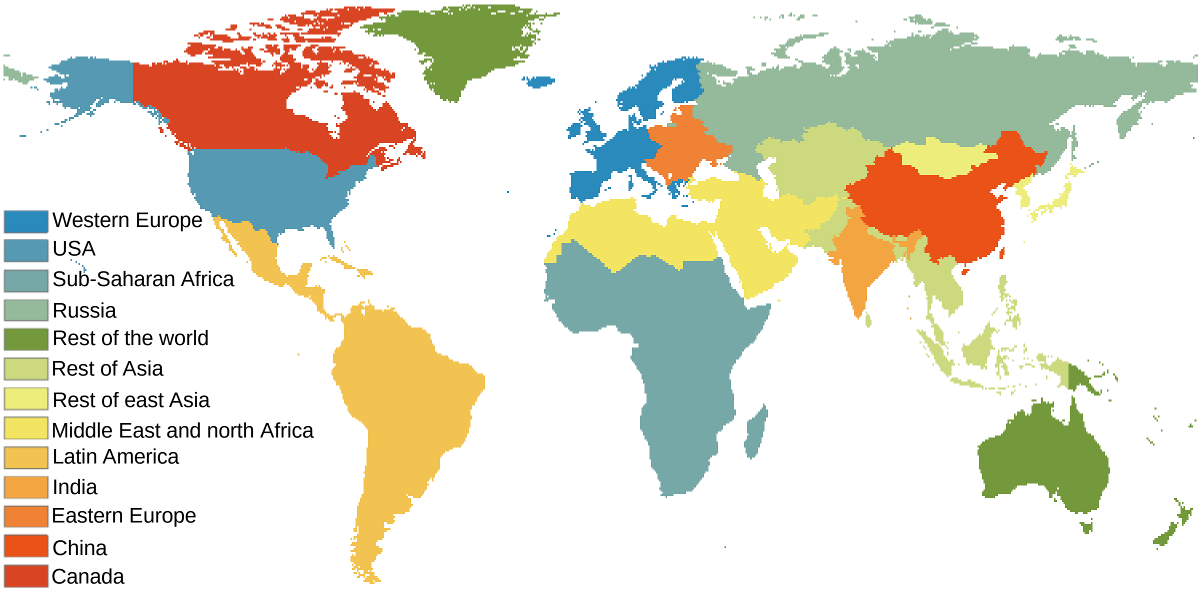

Supplementary Figure 11. The simulated concentration ($ng \cdot m^{-3}$) of B*a*P from the GEOS-Chem model at $1° \times 1°$ resolution, **a**:2012, **b**: 2013, **c**: 2014, **d**: 2015.

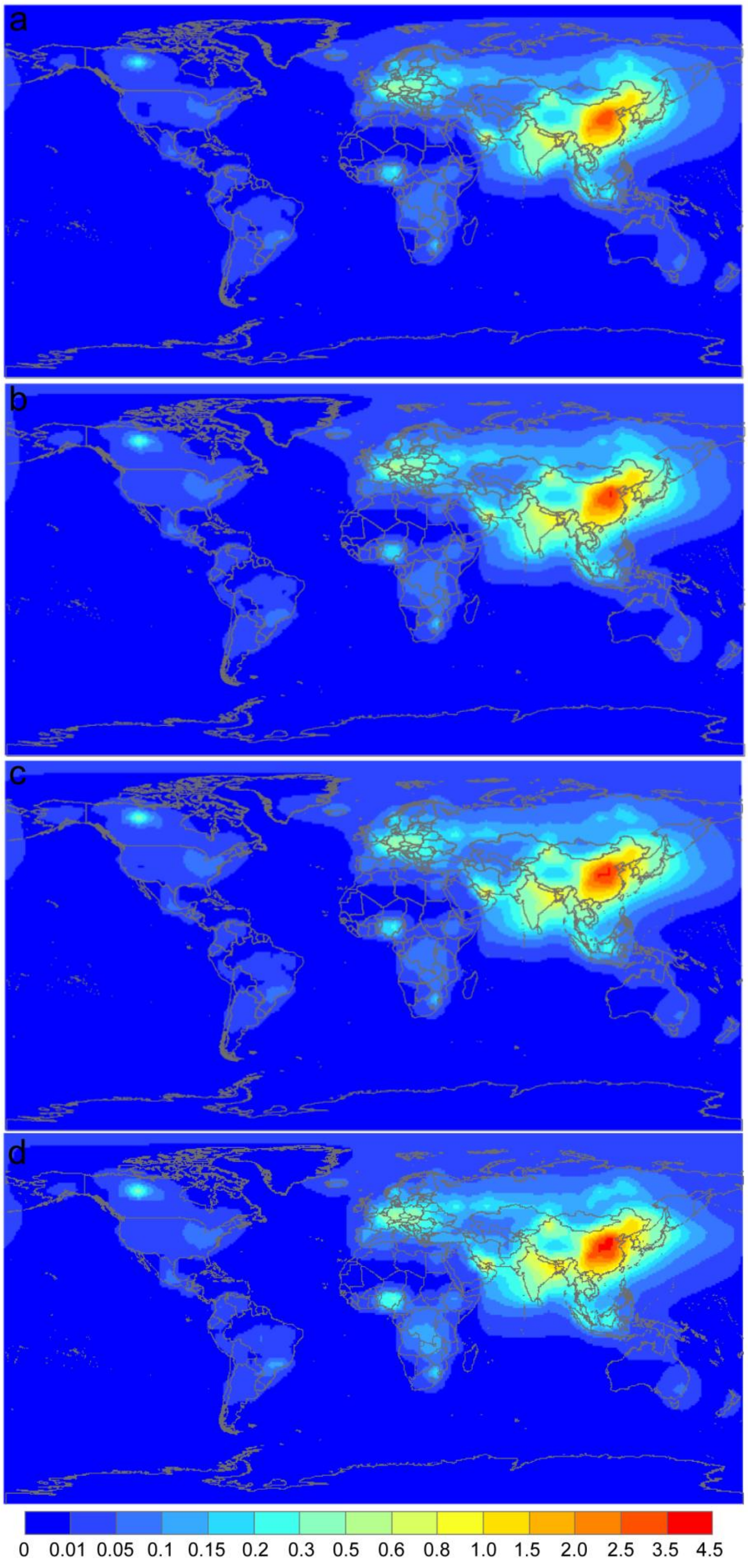

Supplementary Figure 12. The locations of the observations from EMEP, NAPS, and previous studies.

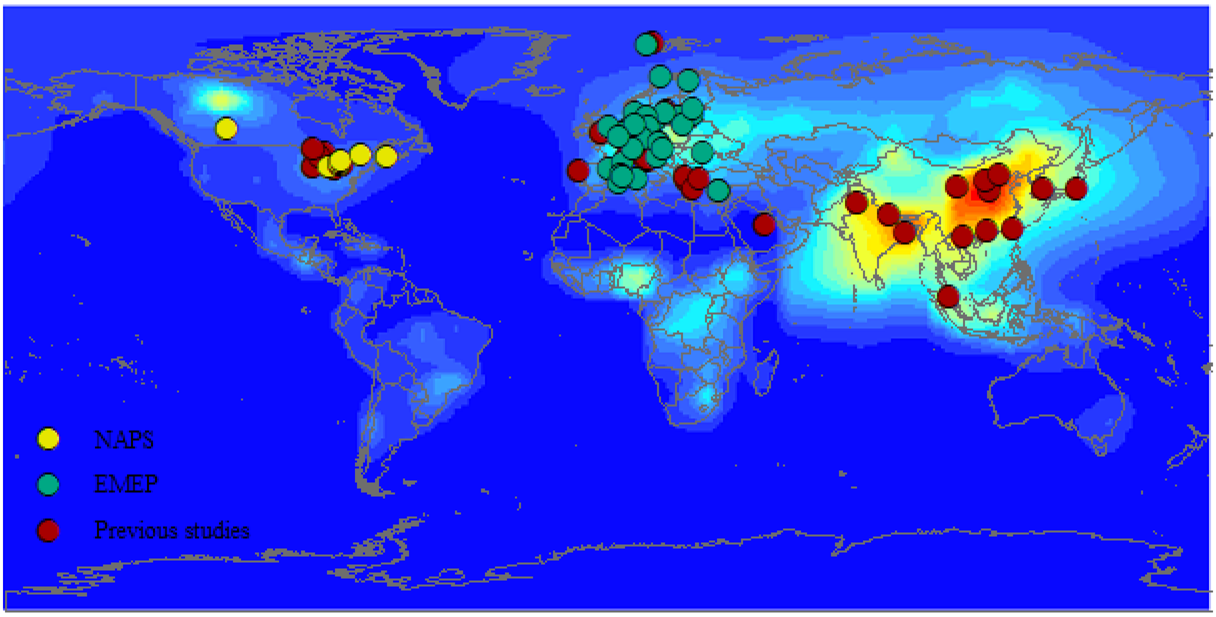

Supplementary Figure 13. The comparison between the simulated values and observations, (a) EMEP, (b) NAPS, (c) references, (d) total.

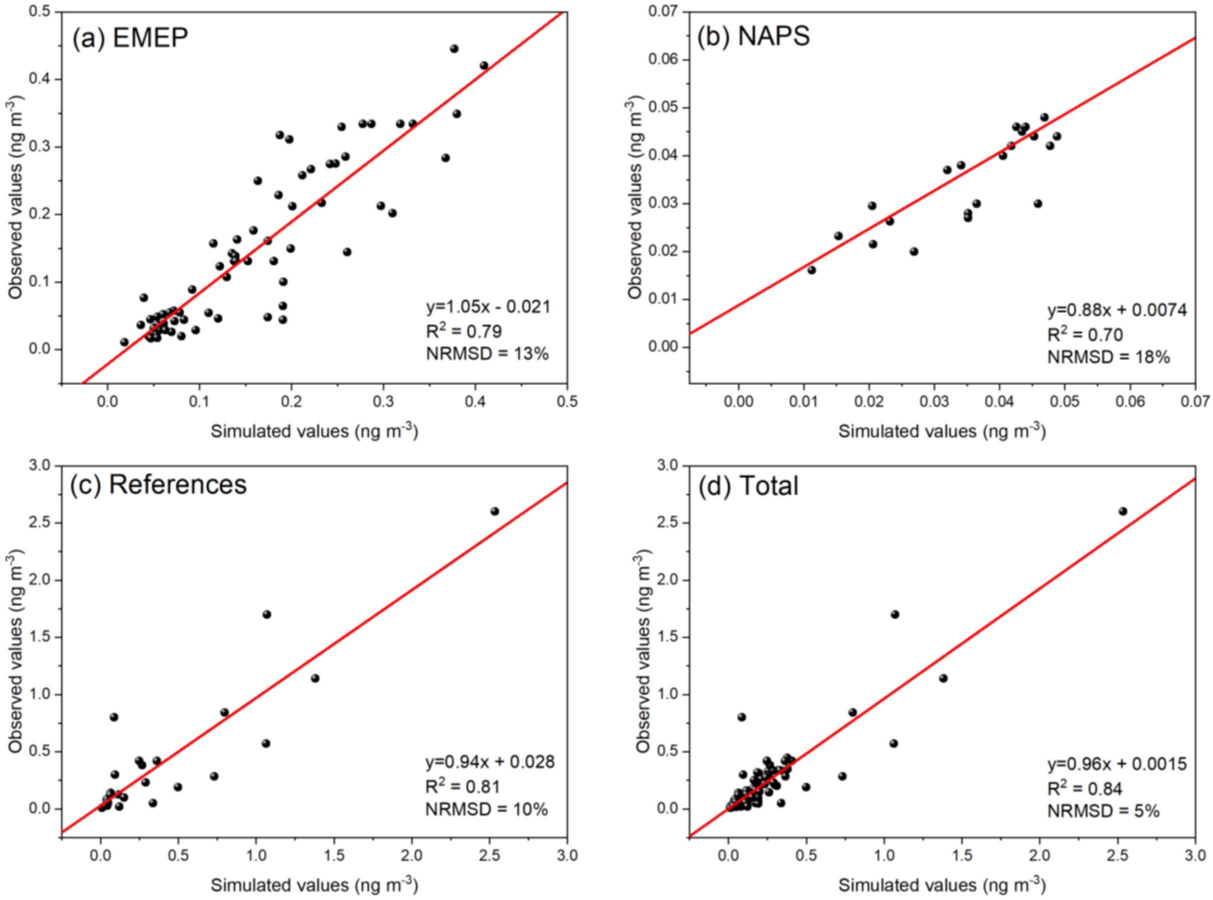

Supplementary Table 1. Parameters of the monovariate regression models for estimating emission factors [1,24]

PAH	NAP	ACY	ACE	FLU	PHE	ANT	FLUH	PYR	BAA	CHR	BBF	BKF	BAP	IDP	DBA	BGP
**ON-ROAD MOTOR VEHICLES**																
K_I	-0.0501	-0.0594	-0.0391	-0.0543	-0.0636	-0.0642	-0.0658	-0.0584	-0.0575	-0.0584	-0.0501	-0.0635	-0.0581	-0.0572	-0.0722	-0.0605
C_I	5.16	3.93	2.92	3.67	4	3.5	3.41	3.46	2.48	2.56	2.31	2.42	2.42	2.26	1.76	2.54
**P**	$1.6*10^{-8}$	$4.3*10^{-9}$	$1.6*10^{-5}$	$<10^{-10}$	$<10^{-10}$	$<10^{-10}$	$<10^{-10}$	$<10^{-10}$	$<10^{-10}$	$<10^{-10}$	$<10^{-10}$	$<10^{-10}$	$<10^{-10}$	$<10^{-10}$	$<10^{-10}$	$<10^{-10}$
R^2	0.21	0.237	0.142	0.266	0.344	0.307	0.402	0.305	0.241	0.313	0.252	0.323	0.317	0.265	0.416	0.32
**SHIPS**																
C_I	2.13	0.39	0.49	0.33	1.13	0.29	-0.11	0.54	-0.4	-0.15	-0.68	-0.5	-0.37	0.07	0.21	-0.31
**AIRCRAFTS**																
C_I	5.16	3.93	2.92	3.67	4	3.5	3.41	3.46	2.48	2.56	2.31	2.42	2.42	2.26	1.76	2.54

Note:

For ships and aircrafts, the coefficients ki from motor vehicles were used directly.

Supplementary Table 2. Parameters of technology split method [1,24]

Sources	Technologies	Ratio description	China				The United States and Canada				Other developed countries				Other developing countries			
			Y_0	Y_f	t_0	s	Y_0	Y_f	t_0	s	Y_0	Y_f	t_0	s	Y_0	Y_f	t_0	s
Energy	a. uncontrol<br>b. control	a/(a+b)	1	0	1979	18	1	0	1907	35	1	0	1915	40	1	0	1945	31
Industry	a. uncontrol<br>b. control	a/(a+b)	1	0	1979	25	1	0	1907	41	1	0	1915	55	1	0	1945	52
Commercial and residential: biomass	a. fireplace<br>b. traditional woodstove<br>c. improved woodstove	c/(b+c)<br>a/(a+b+c)	1	0	1985	23	1	0	1988	21	1	0	1985	20	1	0	1985	23

*Beehive coke production beyond 2010 was assumed to be 0. The fractions of beehive in China have been reported annually since 1949 (China Energy Group. China Energy Databook Version 7.0; Lawrence Berkeley Lab. and Energy Res. Inst.: Berkeley, California, 2008.)

Supplementary Table 3: The conceptual framework of the environmental extended multiregional input-output table (EE-MRIO) in this study.

**Multiregional input-output table**			**Intermediate outputs**							**Final demand**			**Final sale**	**Total output**
			Region 1			…	Region n			Region 1	…	Region n		
			Sector 1	…	Sector j	…	Sector 1	…	Sector j					
**Intermediate inputs**	Region 1	Sector 1	Z_{11}^{11}	…	Z_{1j}^{11}	…	Z_{11}^{1n}	…	Z_{1j}^{1n}	f_1^{11}	…	f_1^{1n}	$\sum_n f_1^{1n}$	X_1^1
		…	…	…	…	…	…	…	…	…	…	…	…	…
		Sector i	Z_{i1}^{11}	…	Z_{ij}^{11}	…	Z_{i1}^{1n}	…	Z_{ij}^{1n}	f_i^{11}	…	f_i^{1n}	$\sum_n f_i^{1n}$	X_i^1
	…	…	…	…	…	…	…	…	…	…	…	…	…	…
	Region m	Sector 1	Z_{11}^{m1}	…	Z_{1j}^{m1}	…	Z_{11}^{mn}	…	Z_{1j}^{mn}	f_1^{m1}	…	f_1^{mn}	$\sum_n f_1^{mn}$	X_1^m
		…	…	…	…	…	…	…	…	…	…	…	…	…
		Sector i	Z_{i1}^{m1}	…	Z_{ij}^{m1}	…	Z_{i1}^{mn}	…	Z_{ij}^{mn}	f_i^{m1}	…	f_i^{mn}	$\sum_n f_i^{mn}$	X_i^m
**Primary inputs (Added values)**			v_1^1	…	v_j^1	…	v_1^n	…	v_j^n					
**Total input**			X_1^1	…	X_j^1	…	X_1^n	…	X_j^n					

Supplementary Table 4. The net flow patterns of PAH emissions and lifetime lung cancer deaths from the perspectives of primary inputs, final sales, and final consumption in each region in 2012 and 2015

Year	Indicators	Economic activity	CA	CH	EE	IN	LA	MN	RA	RE	RW	RU	SS	US	WE	Total net flow
**2012**	Emission net inflow (Gg)	Income-based	0.4	-8.6	-2.6	-4.9	0.7	0.6	-1.5	4.3	1.5	0.9	-5.0	3.6	10.6	22.0
		Final sale-based	0.4	-7.2	-0.3	-2.5	0.3	-0.4	-0.8	3.5	0.9	0.6	-9.2	5.6	9.0	20.3
		Consumption-based	0.6	-12.6	1.1	-4.2	0.4	0.6	-2.4	5.1	1.6	0.8	-12.0	9.5	11.5	28.8
	Deaths net inflow (10^3)	Income-based	0.2	-7.5	0.1	0.6	0.6	0.6	0.1	1.1	0.5	0.5	0.3	0.7	2.1	7.3
		Final sale-based	0.3	-6.6	0.2	0.7	0.5	0.5	0.2	0.4	0.3	0.2	0.1	1.5	2.0	6.4
		Consumption-based	0.3	-10.0	0.3	0.6	0.7	0.8	0.3	0.8	0.5	0.2	0.2	2.7	2.6	10.0
**2015**	Emission net inflow (Gg)	Income-based	0.3	-4.8	-1.8	-4.3	-0.4	0.2	-1.4	2.9	1.3	0.5	-4.3	3.1	8.7	17.0
		Final sale-based	0.4	-5.8	-0.2	-2.3	0.3	-0.7	-0.9	3.7	0.7	0.2	-10.0	5.7	9.0	19.9
		Consumption-based	0.6	-10.2	0.8	-4.0	0.3	0.2	-2.6	5.1	1.3	0.4	-12.4	9.2	11.3	26.5
	Deaths net inflow (10^3)	Income-based	0.2	-7.3	0.1	0.7	0.6	0.7	-0.3	0.9	0.6	0.6	0.3	0.8	2.2	7.7
		Final sale-based	0.3	-8.3	0.3	0.8	0.6	0.7	0.0	0.4	0.3	0.2	0.2	1.9	2.7	8.3
		Consumption-based	0.5	-12.5	0.4	0.6	0.9	1.1	0.1	0.9	0.6	0.3	0.3	3.5	3.4	12.5

Supplementary Table 5. The sector classifications of fuel consumption statistics (IEA) and MRIOT in this study

**MRIOT**	**Fuel consumption statistics (IEA)**	**Sectors in this study**
Agriculture	Agriculture/forestry	Agriculture
Fishing	Fishing	Fishing
Mining and Quarrying	Mining and Quarrying	Mining and Quarrying
Food & Beverages	Food and tobacco	Food & Beverages
Textiles and Wearing Apparel	Textile and leather	Textiles and Wearing Apparel
Wood and Paper	Wood and wood products	Wood and Paper
Petroleum, Chemical and Non-Metallic Mineral Products	Chemical and petrochemical<br>Non-metallic minerals	Petroleum, Chemical and Non-Metallic Mineral Products
Metal Products	Iron and steel<br>Chemical and petrochemical	Metal Products
Electrical and Machinery	Machinery	Electrical and Machinery
Transport Equipment	Transport equipment	Transport Equipment
Other Manufacturing	Industry not elsewhere specified	Other Manufacturing
Electricity, Gas and Water	Main activity producer electricity plants<br>Autoproducer electricity plants<br>Main activity producer CHP plants<br>Autoproducer heat plants<br>Energy industry own use	Electricity, Gas and Water
Construction	Construction	Construction
Transport	Domestic aviation<br>Road<br>Rail<br>Pipeline transport<br>Domestic navigation<br>Transport not elsewhere specified	Transport
Financial Intermediation and Business Activities	Commercial and public services	Commercial and public services

Recycling		
Public Administration		
Maintenance and Repair		
Wholesale Trade		
Retail Trade		
Hotels and Restaurants		
Education, Health and Other Services		
Post and Telecommunications		
Private Households	Residential	Private Households
Others	Final consumption not elsewhere specified	Others
Re-export & Re-import		

Supplementary Table 6. The summary of emissions and lifetime lung cancer deaths occurred in each region from 2012 to 2015

Indicators	Regions	2012	2013	2014	2015
**Emissions**	CA	1.6	1.4	1.6	1.6
**(Gg)**	CH	81.5	80.9	77.9	77.7
	EE	10.4	9.5	9.2	9.1
	IN	50.5	48.3	47.7	47.2
	LA	24.4	25.0	24.0	24.5
	MN	18.6	19.7	20.2	19.9
	RA	44.5	43.7	44.2	42.0
	RE	2.9	2.9	2.8	2.8
	RW	1.7	1.6	1.5	1.5
	RU	5.0	4.0	5.0	5.3
	SS	54.1	57.1	56.3	56.1
	US	5.0	5.1	5.6	4.7
	WE	12.8	12.7	11.3	11.5
	World	313.0	312.1	307.4	303.9
**Deaths**	CA	14	14	14	14
	CH	42881	49556	52967	60200
	EE	165	180	164	175
	IN	2250	2651	2700	3187
	LA	61	83	77	96
	MN	314	371	438	510
	RA	2175	2676	2789	3085
	RE	1130	1347	1442	1413
	RW	8	7	8	8
	RU	51	56	53	54
	SS	79	65	66	90
	US	107	135	141	139
	WE	314	398	356	375
	World	49548	57539	61216	69346

Supplementary References

1 Shen, H. *et al.* Global atmospheric emissions of polycyclic aromatic hydrocarbons from 1960 to 2008 and future predictions. *Environ Sci Technol* **47**, 6415-6424, doi:10.1021/es400857z (2013).

2 Zhang, Y. & Tao, S. Global atmospheric emission inventory of polycyclic aromatic hydrocarbons (PAHs) for 2004. *Atmospheric Environment* **43**, 812-819 (2009).

3 Lenzen, M. Environmentally important paths, linkages and key sectors in the Australian economy. *Structural Change and Economic Dynamics* **14**, 1-34 (2003).

4 Nagashima, F., Kagawa, S., Suh, S., Nansai, K. & Moran, D. Identifying critical supply chain paths and key sectors for mitigating primary carbonaceous PM2. 5 mortality in Asia. *Economic Systems Research* **29**, 105-123 (2017).

5 Chang, N. & Lahr, M. L. Changes in China's production-source CO2 emissions: insights from structural decomposition analysis and linkage analysis. *Economic Systems Research* **28**, 224-242 (2016).

6 Zhang, Y. Supply-side structural effect on carbon emissions in China. *Energy Economics* **32**, 186-193 (2010).

7 Chen, L. *et al.* Trans-provincial health impacts of atmospheric mercury emissions in China. *Nat Commun* **10**, 1484, doi:10.1038/s41467-019-09080-6 (2019).

8 Zhang, H. *et al.* Impacts of supply and consumption structure on the mercury emission in China: An input-output analysis based assessment. *Journal of Cleaner Production* **170**, 96-107, doi:10.1016/j.jclepro.2017.09.139 (2018).

9 Li, R., Zhang, J. & Krebs, P. Consumption- and Income-Based Sectoral Emissions of Polycyclic Aromatic Hydrocarbons in China from 2002 to 2017. *Environ Sci Technol* **55**, 3582-3592, doi:10.1021/acs.est.0c08119 (2021).

10 Zhang, Q. *et al.* Transboundary health impacts of transported global air pollution and international trade. *Nature* **543**, 705-709, doi:10.1038/nature21712 (2017).

11 Wiedmann, T., Wilting, H. C., Lenzen, M., Lutter, S. & Palm, V. Quo Vadis MRIO? Methodological, data and institutional requirements for multi-region input–output analysis. *Ecological Economics* **70**, 1937-1945 (2011).

12 Steen-Olsen, K. *et al.* Accounting for value added embodied in trade and consumption: an intercomparison of global multiregional input–output databases. *Economic Systems Research* **28**, 78-94 (2016).

13 Lenzen, M., Wood, R. & Wiedmann, T. Uncertainty analysis for multi-region input–output models–a case study of the UK's carbon footprint. *Economic Systems Research* **22**, 43-63 (2010).

14 Peters, G. P., Davis, S. J. & Andrew, R. A synthesis of carbon in international trade. *Biogeosciences* **9**, 3247-3276 (2012).

15 Lin, J. *et al.* Global climate forcing of aerosols embodied in international trade. *Nature Geoscience* **9**, 790-794 (2016).

16 Armstrong, B., Hutchinson, E., Unwin, J. & Fletcher, T. Lung cancer risk after exposure to polycyclic aromatic hydrocarbons: a review and meta-analysis. *Environ Health Perspect* **112**, 970-978, doi:10.1289/ehp.6895 (2004).

17 Mi, Z. *et al.* Chinese CO2 emission flows have reversed since the global financial crisis. *Nat Commun* **8**, 1712, doi:10.1038/s41467-017-01820-w (2017).

18 Shrivastava, M. *et al.* Global long-range transport and lung cancer risk from polycyclic aromatic

hydrocarbons shielded by coatings of organic aerosol. *Proc Natl Acad Sci U S A* **114**, 1246-1251, doi:10.1073/pnas.1618475114 (2017).

19 Zhou, S. *et al.* Multiphase reactivity of polycyclic aromatic hydrocarbons is driven by phase separation and diffusion limitations. *Proceedings of the National Academy of Sciences* **116**, 11658-11663 (2019).

20 Singh, A. *et al.* Assessing hazardous risks of indoor airborne polycyclic aromatic hydrocarbons in the kitchen and its association with lung functions and urinary PAH metabolites in kitchen workers. *Clinica chimica acta* **452**, 204-213 (2016).

21 Ohura, T., Amagai, T., Fusaya, M. & Matsushita, H. Polycyclic aromatic hydrocarbons in indoor and outdoor environments and factors affecting their concentrations. *Environmental science & technology* **38**, 77-83 (2004).

22 Hong, C. *et al.* Global and regional drivers of land-use emissions in 1961–2017. *Nature* **589**, 554-561 (2021).

23 Zhu, E. *et al.* Carbon emissions induced by land-use and land-cover change from 1970 to 2010 in Zhejiang, China. *Science of the Total Environment* **646**, 930-939 (2019).

24 Shen, H. *et al.* Global time trends in PAH emissions from motor vehicles. *Atmospheric Environment* **45**, 2067-2073 (2011).
