# Supplementary material for: Global trade drives transboundary transfer of the health impacts of polycyclic aromatic hydrocarbon emissions
Source: Commun Earth Environ. 2022 Aug 1;3(1):170. doi: 10.1038/s43247-022-00500-y (PMC9340739; doi:10.1038/s43247-022-00500-y)
Supplement: Supplementary file 9 — Description of Additional Supplementary Files [file 43247_2022_500_MOESM9_ESM.pdf]

Description of Additional Supplementary Files

File Name: Supplementary Data 1

Description: The summary of data sources

File Name: Supplementary Data 2

Description: The emission factors for different sources

File Name: Supplementary Data 3

Description: The contributions of different drivers to the changes in PAH emissions (Gg) in 13 worldwide regions from 2012 to 2015

File Name: Supplementary Data 4

Description: The contributions of different drivers to the changes in the lifetime lung cancer deaths in 13 worldwide regions from 2012 to 2015.

File Name: Supplementary Data 5

Description: The scenarios applied in EE-MRIO and SDA methods

File Name: Supplementary Data 6

Description: The 190 regions in Eora input output table and corresponding 13 regions in this study

File Name: Supplementary Data 7

Description: The comparison between the simulated values and observations ($\mathrm{ng \cdot m^{-3}}$) from EMEP, NAPS, and previous studies
